# Characterization, genomics, and applications of the *Cronobacter sakazakii* bacteriophage Csp-D17

**DOI:** 10.1128/spectrum.00731-25

**Published:** 2025-11-11

**Authors:** Yue-yue Zhang, Ding-rong Zhang, Zhen-quan Yang, Lei Yuan, Ya Zhao, Yuan-song Zhang

**Affiliations:** 1 Department of Cardiology, Shanghai Yangpu District Kongjiang Hospital 668785 , Shanghai, China; 2 Department of Neurosurgery, Chongqing Municipal Corps Hospital of Chinese People’s Armed Police Force, Chongqing, China; 3 School of Food Science and Engineering, Yangzhou University 38043, Yangzhou, Jiangsu, China; 4 Department of Nutrition, Shanghai East Hospital, Tongji University School of Medicine 481875, Shanghai, China; MultiCare Health System, Tacoma, Washington, USA

**Keywords:** *Cronobacter sakazakii*, bacteriophage, genomics, biocontrol agent

## Abstract

*Cronobacter sakazakii* is an opportunistic pathogen that can contaminate infant formula, resulting in meningitis and neurological disorders in infants. Bacteriophages have been consistently identified as effective inhibitors of pathogenic bacteria. In this study, a lytic phage, Csp-D17, was isolated from environmental wastewater, and its characterization, genomics, and application were analyzed. Transmission electron microscopy revealed that the phage belonged to the order *Caudoviricetes* within the family *Ackermannviridae*. The phage Csp-D17 exhibited notable stability across varied thermal and pH conditions, with an optimal multiplicity of infection of 0.01 and a lysis capacity of 40 plaque-forming units per infected cell. Evaluation of its host range revealed lytic activity against 4 out of 11 tested strains of *C. sakazakii* (36.4%). The frequency of insensitive mutations in *C. sakazakii* D17 to phage Csp-D17 was 3.8 × 10^−7^. High-dose phage treatment was more effective in inhibiting the growth of *C. sakazakii* D17 in the powdered infant formula model, which was correlated with its short latency period, high lysis amount*,* and low frequency of insensitive mutations in the host bacteria. The results demonstrated that phage Csp-D17 was not lysogenic. Furthermore, the genomic study of phage Csp-D17 revealed that the genome structure of this phage was a double-stranded DNA molecule with a length of 143,081 base pairs, a guanine + cytosine content of 46.42%, 233 open reading frames (the average gene length is 553 base pairs), and it does not contain any genes related to virulence or lysogenicity. Furthermore, the genetic function, expression mechanism*,* and phylogenetic diversity of Csp-D17 were analyzed by genomic comparison. This study provides a theoretical basis for developing novel biocontrol agents based on phage control of *C. sakazakii*.

**IMPORTANCE:**

This study significantly advances the field of food safety biocontrol by identifying and characterizing Csp-D17, a novel lytic phage specifically targeting *Cronobacter sakazakii*. Through comprehensive morphological, physiological, and whole-genomic analyses, Csp-D17 is rigorously confirmed as a distinct and well-defined viral agent. The demonstration that Csp-D17 can serve as a potent biocontrol agent against *C. sakazakii* in powdered infant formula highlights its direct practical application in mitigating a critical pathogen responsible for severe neonatal infections. This work not only contributes a new tool to the antimicrobial arsenal but also provides a foundational framework for developing phage-based strategies to enhance the safety of food products, particularly those intended for vulnerable populations.

## INTRODUCTION

*Cronobacter sakazakii* is an opportunistic pathogen (1). Given that contaminated powdered infant formula (PIF) is the most common source of infection, infants are at high risk of *C. sakazakii* infection, which can lead to meningitis and other neurological diseases (2, 3). Since the initial report of *C. sakazakii* infection in Tennessee, USA, in April 2001, the pathogen has posed a significant threat to infant health (4). A notable instance was the 2022 contamination of PIF at Abbott Nutrition in the USA, which tragically led to the deaths of four infants and young children (5). In addition, a concerning study from China revealed an 18.3% prevalence of *C. sakazakii* in samples from a PIF plant, encompassing both environmental isolates and a PIF sample itself (6). Currently, the management of *C. sakazakii* infections predominantly relies on antibiotic therapy. However, the efficacy of these treatments is increasingly being undermined by the rising incidence of antibiotic resistance within *C. sakazakii* populations (7). Given the potentially lethal consequences of *C. sakazakii* infection in infants, it is imperative to prioritize the prevention and control of this pathogen in PIF production. Therefore, identifying viable and sustainable therapeutic or prophylactic alternatives represents an urgent research priority.

Bacteriophages (phages) are obligate intracellular viruses that specifically target and infect bacteria (8). They share several common characteristics of viruses, including small size, lack of a cellular structure, the ability to lyse and replicate within living bacterial hosts, and an incredibly high abundance in the environment (especially in environmental wastewater), estimated to be between 10^30^ and 10^32^ particles (9). It is now understood that phages infect bacteria by relying on their characteristic tail adsorption to infect the bacteria, injecting their nucleic acid, and synthesizing phages. Newly assembled phage particles are then released through bacterial lysis, resulting in host cell death (10–12). The specific recognition-lysis mechanism between the phage tail and host can be exploited as a natural and novel biocontainment agent (13). For instance, the phage isolated by Lee et al. (14) suppressed the growth of *C. sakazakii* within 2 h. Furthermore, the phage exhibited rapid inhibitory effects on *C. sakazakii* growth. Wang et al. (15) isolated and obtained a lytic *C. sakazakii* phage, designated JK004, which exhibited potent inhibitory activity against *C. sakazakii* on material surfaces and in PIF. Jiang et al. (16) isolated phage JC01 that demonstrated high stability under diverse environmental conditions and lacked potential virulence genes. Furthermore, it exhibited the capacity to lyse *C. sakazakii* in PIF. While the authors demonstrated the biocontrol ability of their *C. sakazakii* phages with unique lysis properties, they did not report full gene sequencing and related bioinformatics studies before application. Consequently, current reports provide an incomplete understanding of *C. sakazakii* phages at the molecular level. To ensure the safety of phage therapy applications, whole-genome sequencing is essential to exclude phages harboring virulence and lysogeny determinants, thereby preventing the dissemination of virulence genes.

Accordingly, a phage specific to *C. sakazakii* was isolated from environmental wastewater collected in Yangzhou. The isolated phage was then subjected to a two-pronged analysis: biological characterization and bioinformatics analysis. Finally, to assess the phage’s inhibitory potential against *C. sakazakii* in a relevant food product, an artificial contamination model was constructed using PIF. Overall, this isolated, novel phage exhibits potential for application in PIF-related biocontrol strategies targeting *C. sakazakii*.

## MATERIALS AND METHODS

### Bacterial strains and culture conditions

Eleven *C. sakazakii* strains (listed in Table S1) were inoculated into Nutrient Broth (NB) and incubated at 37°C for 12 h in a shaking incubator (HZ-8812SB, Hualida Co., Ltd., Suzhou, China) at a rotational speed of 120 rpm. *Escherichia coli* 10664, *Salmonella* Enteritidis 21513, *Staphylococcus aureus* 21600, *Bacillus cereus* 21261, *Enterobacter hormaechei* YZU2-5, *Listeria monocytogenes* 19111, *Vibrio parahaemolyticus* YZU84, and *Brochothrix thermosphacta* YZU-2-7 (Table S1) were cultured at 37°C for 12 h with shaking at 120 rpm in Luria-Bertani medium (Huankai Microbial Sci & Tec. Co. Ltd., Guangzhou, China).

### Isolation, purification, and concentration of *C. sakazakii* phage

Phage isolation and enrichment from sewage samples were performed according to the protocol described by Chen et al. (17), involving centrifugation (8,000 rpm, 10 min) and filtration through a 0.22 µm membrane. Phage purification was performed via the double-layer agar plating method as documented by Yang et al. (18). Individual plaques were excised and suspended in SM buffer (8 mM MgSO_4_∙7H_2_O, 100 mM NaCl, 0.02% gelatin, 50 mM Tris-HCl, pH 7.5), followed by three rounds of plaque assay purification. Phage titration was conducted using the double-layer agar method (19). The phage titers (PFU/mL) were calculated using (equation 1):


(1) $$Titer=\frac{\bar{x}}{0.1\times d}$$

where titer is the phage potency (PFU/mL), $\bar{x}$ is the average phage vacuole number (PFU), 0.1 is the inoculum volume (mL), and *d* is the sample dilution. Phage concentration and purification were adapted from the approach reported by Tang et al. (20) as follows: (i) nuclease treatment (DNase I/RNase A, 1 µg/mL each); (ii) PEG 8000 precipitation (10% wt/vol, 5 h on ice); (iii) CsCl density gradient ultracentrifugation (1.45–1.7 g/mL, 30,000 rpm, 2 h). Detailed protocols for *C. sakazakii* bacteriophage isolation, purification, and concentration are displayed in Supporting Information 1.1.

### Characterization of isolated phage

#### Micromorphological observation

Phage morphology was examined using a Tecnai-12 transmission electron microscope (TEM, Thermo Fisher Scientific, Eindhoven, Netherlands) at an acceleration voltage of 120 kV, with micrographs acquired at magnifications ranging from ×30,000 to ×50,000. Sample preparation followed the method described by Byun et al. (21) with minor modifications. Briefly, 10 µL of 2% phosphotungstic acid (PTA, Aladdin Biochemical Technology Co., Ltd., Shanghai, China) was added dropwise to 30 µL of concentrated phage solution. The mixture was incubated for 10 min to allow staining. Excess PTA was removed by gently blotting with filter paper. The sample was then air-dried with an infrared lamp at room temperature for 30 min.

Scanning electron microscopy (SEM) was employed to investigate the effect of phage treatment on the morphology of *C. sakazakii* D17. A GeminiSEM 300 instrument (Carl Zeiss, Germany) was used for this analysis. The experiment was adapted from a previously described method (22). Briefly, 5 mL of NB culture medium was added to each well of a 24-well plate. A sterilized climbing piece, sized slightly smaller than the well diameter, was placed at the bottom of each well. Subsequently, 800 µL of a *C. sakazakii* D17 suspension (adjusted to a concentration of 10^8^ colony-forming units [CFU]/mL) and 200 µL of phage solution (10^9^ PFU/mL) were added to each well. The plates were incubated without agitation at 37°C for 12 h. Following incubation, the supernatant was removed, and the wells were washed twice with phosphate-buffered saline (pH 7.5) to eliminate unattached bacterial cells. For SEM sample preparation, the cell monolayers were fixed with 2.5% (wt/vol) glutaraldehyde and dehydrated through a graded ethanol series (30%, 50%, 70%, 80%, 90%, 95%, and 100%), followed by treatment with 100% anhydrous sodium sulfate. The dehydrated samples were then subjected to critical point drying (CPD-300) and subsequently sputter-coated with gold (30 s, 20 mA) using an ion sputtering apparatus (SCD 500) for SEM analysis at an accelerating voltage of 20 keV.

#### Determination of phage lysis spectrum

The lysis spectrum was determined using the spotting method described by Zhang et al. (19). A total of 10 µL of a bacterial suspension (adjusted to a concentration of 10^8^ CFU/mL) was spotted onto an NB agar plate. Following air-drying, the plate was incubated at 37°C for 4 h to allow complete formation of a uniform bacterial lawn. Two microliters of the phage solution (at a concentration of 10^9^ PFU/mL) was then spotted onto the corresponding bacterial spot. The plate was again air-dried and incubated at 37°C for 30 min. A clear lysis zone surrounding the phage droplet indicated the phage’s ability to lyse the specific bacterial strain. The bacterial strains used in this assay (*n* = 19) are listed in Table S2.

#### Lysogenicity analysis

To assess the presence of lysogenic conversion in the isolated phage, a double-layer agar assay was employed, with slight modifications based on the methods described by Li et al. (23) and Khemayan et al. (24). Briefly, 100 µL of a host bacterial culture (adjusted to a concentration of 10^8^ CFU/mL) was spread evenly onto the surface of an NB agar plate. The plate was allowed to air-dry completely to form a uniform bacterial lawn. Next, 50 µL of the phage amplification solution (at a concentration of 10^9^ PFU/mL) was carefully dropped onto the solidified agar such that it intersected with the bacterial lawn at a 90° angle. The plate was again air-dried to allow complete absorption. Following incubation at 37°C for 8 h, the plate was examined for bacterial growth at the intersection of the phage droplet and the bacterial lawn. The absence of bacterial colonies at the intersection indicated a lack of lysogenic conversion by the phage.

#### Thermal and pH stability test

The thermal stability of the phage was determined based on the method described by Tang et al. (25). Two milliliters of phage solution (at a concentration of 10^9^ PFU/mL) was incubated at each of the following temperatures: 50°C, 60°C, 70°C, and 80°C for 60 min. Aliquots of 500 µL were withdrawn at 0, 20, 40, and 60 min, and the phage titer was determined using the double-layer agar plate method. To assess the phage’s stability at different pH values, 100 µL of the phage solution (adjusted to a concentration of 10^8^ PFU/mL) was added to separate tubes containing 900 µL of NB medium adjusted to each of the following pH values: 2.0–12.0. The mixtures were incubated at 37°C for 2 h. Phage titer was then determined using the double-layer agar plate method. The pH stability of the phage was expressed as the percentage survival rate, calculated as described in (equation 2).


(2) Phagesurvivalrate=(phagetiterincubatedatdifferentpH) / (phagetiterincubatedatpH7.0)×100%

#### Determination of the optimal multiplicity of infection (MOI)

The optimal MOI for phage infection was determined with slight modifications based on the method described by Cheng et al. (26). Briefly, 100 µL of a host bacterial culture (adjusted to a concentration of 10^5^ CFU/mL) was mixed with 100 µL of phage solution at various ratios (0.001, 0.01, 0.1, 1, and 10 PFU/CFU) in separate tubes containing 5 mL of NB medium. The mixtures were incubated at 37°C for 8 h. Following incubation, the cultures were centrifuged at 8,000 rpm for 10 min at 4°C. The supernatants were collected and passed through a 0.22 µm filter membrane. The MOI that yielded the highest phage titer, as determined by the double-layer agar plate method, was considered the optimal MOI.

#### One-step growth curve experiment

A one-step growth curve was generated to determine the phage burst size following the method described by Choudhary et al. (27) and Kurt et al. (28), with minor modifications. Briefly, 100 µL of the phage solution was mixed with 100 µL of the host bacteria culture (adjusted to the optimal MOI) in 5 mL of NB medium. The mixture was incubated at 37°C for 5 min for phage adsorption. The culture was then centrifuged at 8,000 rpm for 10 min at 4°C to remove unadsorbed phage particles. The phage pellet was subsequently washed three times with fresh NB medium to remove residual free phage particles. After washing, 5 mL of fresh NB medium was added to the phage pellet, and the culture was incubated at 37°C for 120 min. Aliquots of 100 µL culture medium were collected at 5 min intervals for the first 60 min, followed by 20 min intervals for the remaining 60 min. The phage titer in each sample was determined using the double-layer agar plate method. The data obtained were used to construct a one-step growth curve by plotting the infection time (min) on the *x*-axis and the logarithmic value of the phage titer (log PFU/mL) on the *y*-axis. The burst size was calculated as described in (equation 3).


(3) Burstsize=averagephagetiterduringtheburstperiod / averagephagetiterduringthelatentperiod

#### Determination of the frequency of insensitive mutation

The frequency of bacteriophage-insensitive mutants (BIMs) in *C. sakazakii* D17 was determined following the approach documented by Gutiérrez et al. (29) with modifications. Putative BIMs were isolated by mixing 100 µL phage solution (10^8^ PFU/mL) with 100 µL host culture (10^6^ CFU/mL). The experimental group received 50 µL CaCl_2_ and 50 µL MgSO_4_ (both 10 mmol/L), while the control received 100 µL sterile saline. After a 10 min incubation at 37°C, 100 µL aliquots were plated on NB agar and incubated for 18 h at 37°C. Colonies growing exclusively on the experimental plate were considered putative BIMs. These isolates were validated via a standardized spot test. Briefly, cultures were initially isolated by streaking on nutrient agar plates, then cultured in NB broth. A 10 µL sample of each culture was spotted onto an agar plate and then overlaid with 2 µL phage (10^9^ PFU/mL). The plates were incubated for 8 h at 37°C. Isolates showing no lysis zone were confirmed as BIMs. The BIM frequency was calculated using (equation 4):


(4) $$BIM=N/10^{6}$$

where *BIM* is the frequency of insensitive mutations, *N* is the identified insensitive mutant strain, and 10^6^ is the host bacterial concentration (CFU/mL). Detailed protocols for determination of the frequency of insensitive mutation are displayed in Supporting Information 1.2.

### Genomic analysis of phage

Phage genomic analysis was performed as previously described by Zhou et al. (30, 31). Concentrated and purified phage preparations (Supporting Information 1.1) underwent DNA extraction using the λ Phage Genomic DNA Extraction Kit (Tiangen, China). The extracted DNA underwent paired-end sequencing on the Illumina HiSeq 4000 platform (Illumina Inc., USA). Genome assembly and functional annotation were subsequently performed using the resulting sequencing data. Homology analysis of the assembled genome was conducted via BLAST on the NCBI database (https://www.ncbi.nlm.nih.gov/). Genetic maps were generated using IBS 1.0.2, and protein sequence comparisons (>90% identity) between phage strains were visualized with Adobe Illustrator 2022.

### Inhibitory effect of phage on the growth of *C. sakazakii* D17

Next, we sought to investigate the inhibitory effect of a bacteriophage on *C. sakazakii* D17 using the method previously described by Maung et al. (32). Briefly, 100 µL of a *C. sakazakii* D17 inoculum (adjusted to a concentration of 10^4^ CFU/mL) was mixed with 100 µL of the bacteriophage inoculum at various MOIs of 100, 10, 1, 0.1, and 0.01. The control group received no bacteriophage treatment. All samples, including the PIF at a concentration of 12% (wt/vol), were incubated at 37°C for 24 h. The number of colony-forming units (CFU/mL), optical density at 600 nm (OD_600_), and phage titer were measured every 2 h. The maximum bacteriophage inhibition rate (h. Imax) was determined using (equation 5).


(5) $$Imax=(OD_{600}\;of\;the\;control\;group\;-\;OD_{600}\;of\;the\;different\;MOI\;groups)\;/\;OD_{600}\;of\;the\;control\;group\times 100\%$$

### Statistical analysis

All experiments were performed in triplicate, and the data were presented as mean ± standard deviation. Statistical analysis was performed using SPSS 24.0 software (IBM Corp., Armonk, NY, USA). Graphs were generated with Origin Pro 2021 software (OriginLab Corporation, Northampton, MA, USA). Statistical significance was determined using a one-way or two-way ANOVA with Tukey’s multiple comparisons test. A *P*-value <0.05 was considered statistically significant. Different lowercase letters and asterisks indicate significant differences between groups.

## RESULTS

### Isolation and morphological characteristics of phage infecting *C. sakazakii* D17

**Fig 1:**
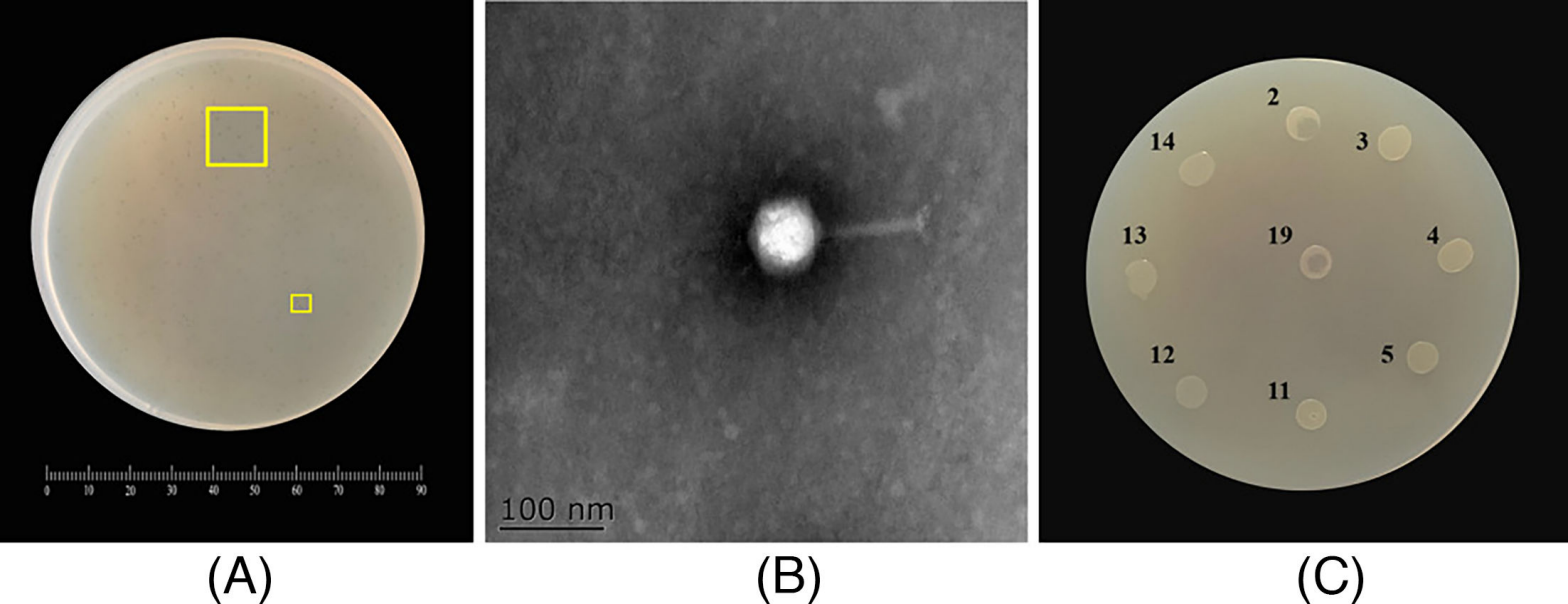
Plaques of Csp-D17 on the *C. sakazakii* D17 plate (Petri dish diameter, 90 mm) (**A**). Electron micrographs of Csp-D17 (**B**), and the partial lysis spectrum of Csp-D17 (**C**): no. 2, *C. sakazakii* 22919, no. 3, *C. sakazakii* 21545, no. 4, *C. sakazakii* 21673, no. 5, *C. sakazakii* 21569, no. 11, *E. coli* 10664, no. 12, *S*. Enteritidis 21513, no. 13, *S. aureus* 21600, no. 14, *B. cereus* 21261, no. 19, *C. sakazakii* D17. Scale bar, 100 nm.

In this study, we investigated bacteriophages that infect *C. sakazakii* D17. A novel phage, Csp-D17, was isolated from wastewater samples collected in Yangzhou, China. This phage demonstrated the ability to effectively lyse the host bacterium *C. sakazakii* D17 (Fig. 1). As shown in Fig. 1A, Csp-D17 forms clear, transparent plaques approximately 0.15 mm in diameter, lacking any opaque halo. TEM analysis revealed that phage Csp-D17 virions possessed isometric polyhedral heads (86 nm in diameter) and contractile tails (107 nm in length) (Fig. 1B). Based on the classification criteria established in the 2022 update of the International Committee on Taxonomy of Viruses for bacterial viruses (33), Csp-D17 belongs to the order *Caudoviricetes* within the family *Ackermannviridae*.

### The phage life cycle

To evaluate the potential of Csp-D17 as a biocontrol agent, a panel of 19 bacterial strains was screened for susceptibility to lysis by this phage. *C. sakazakii* D17, the original host from which Csp-D17 was isolated, served as a positive control in this experiment. As shown in Table S2, Csp-D17 displayed lytic activity against 4 of the 19 tested bacterial strains, specifically targeting the *C. sakazakii* species. Within this taxonomic group, the phage achieved a host range coverage of approximately 36.4% (4 susceptible isolates out of 11 *C*. *sakazakii* strains evaluated). Conversely, Csp-D17 exhibited no lytic activity against the remaining bacterial strains, including *E. coli* 10664, *S*. Enteritidis 21513, *S. aureus* 21600, *B. cereus* 21261, *E. hormaechei* YZU2-5, *L. monocytogenes* 19111, *V. parahaemolyticus* YZU84, and *B. thermosphacta* YZU-2-7 (Fig. 1C).

An additional concern regarding phage application is lysogenicity. Lysogenic phages, also known as temperate phages, integrate their genome into the host bacterium’s chromosome upon infection, becoming a prophage. This prophage does not lyse the host and does not produce progeny phages immediately. However, under certain environmental pressures, the prophage can excise itself from the chromosome and enter the lytic cycle, lysing the host and producing new phage particles (34). Figure S1 demonstrates the lytic activity of Csp-D17 on *C. sakaza*kii D17. The absence of bacterial growth at the intersection of the phage and bacterial cultures in the spot test indicates that Csp-D17 is a virulent phage that directly lyses its host, *C. sakazakii* D17, during infection. The lack of lysogeny observed in this experiment suggests that Csp-D17 may be safer for biocontrol applications.

### pH and thermal stability of phage

**Fig 2:**
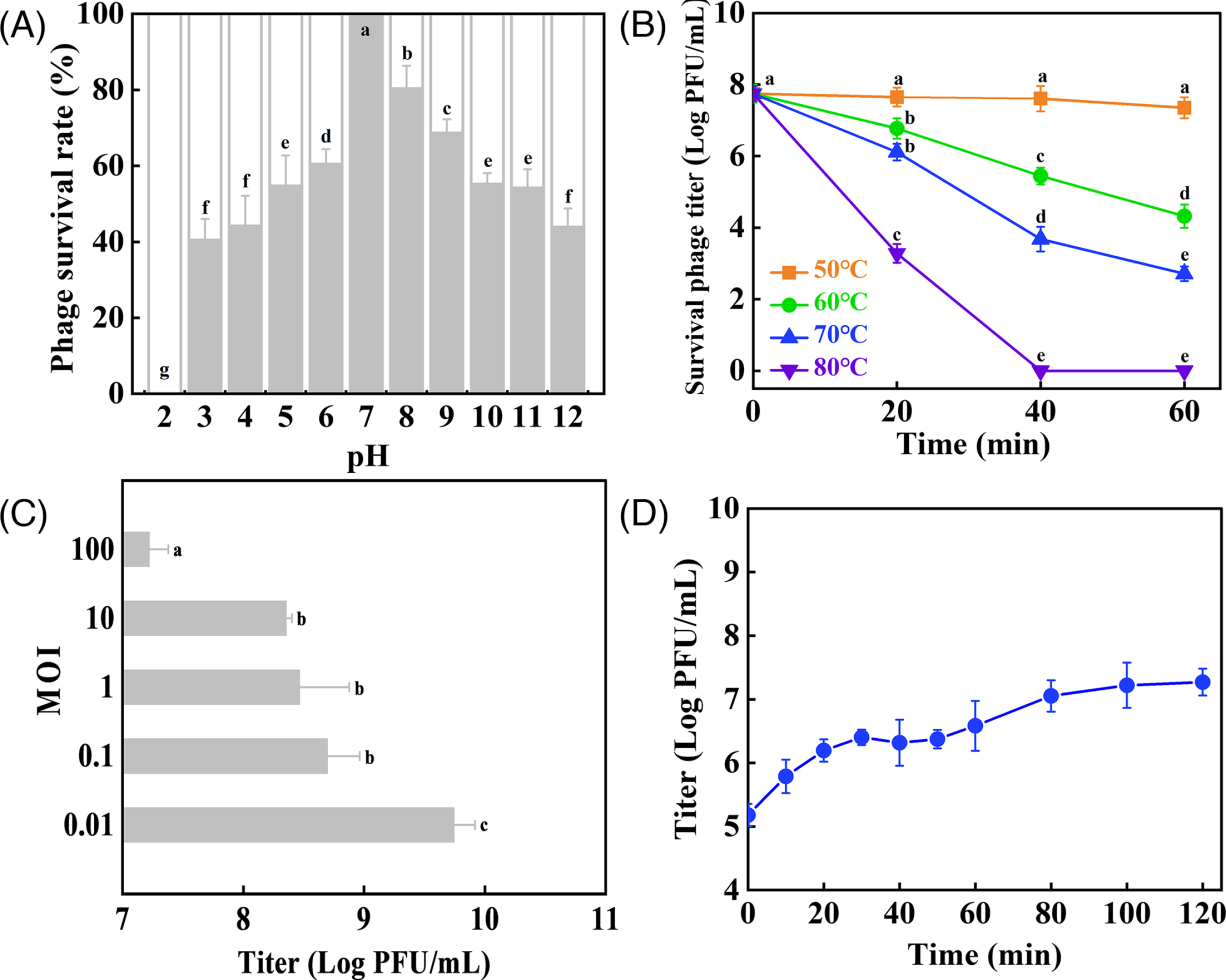
Stability of Csp-D17 under different stress conditions and its biological characterization. (**A**) pH stability: Csp-D17 was incubated under different pH conditions (2–12) for a period of 2 h at 37°C. (**B**) Thermal stability: Csp-D17 was incubated at a range of temperatures (50°C, 60°C, 70°C, and 80°C) under optimal pH conditions, and the titer of surviving phage was measured at 20 min intervals over 60 min. (**C**) Multiplicity of infection: *C. sakazakii* D17 (10^5^ CFU/mL) was infected with Csp-D17 under MOI conditions (100, 10, 1, 0.1, and 0.01) for 8 h. (**D**) One-step growth curve: Csp-D17 suspensions were collected at 5 min intervals for up to 60 min, followed by 20 min intervals for up to 120 min. Different letters indicate significant differences (*P* < 0.05, Tukey’s multiple comparisons test). A one-way ANOVA was applied for panels (**A and C**) and a two-way ANOVA for panel (**B**).

A study has shown that environmental factors such as pH and temperature can significantly impact phage adsorption, infiltration, and proliferation (35). Therefore, evaluating a phage’s tolerance to these conditions is crucial for its potential industrial applications. Figure 2A depicts the effect of pH on the Csp-D17 survival rate. After incubation at 37°C for 2 h, the phage exhibited a varied decrease in viability at pH values outside the range of 6 to 9 (*P* < 0.05). Notably, Csp-D17 maintained over 60% viability within this pH range. However, the phage activity sharply declined at pH values below 2.0. Figure 2B illustrates the thermal stability of Csp-D17. The phage displayed bioactivity of 10^7^ PFU/mL after incubation at 50°C for 60 min. While some activity remained (10^2^–10^4^ PFU/mL) at 60°C and 70°C for 60 min, complete inactivation occurred at 80°C within 40 min.

### Determination of optimal MOI

MOI refers to the ratio of phage particles to host bacterial cells (36). The MOI that yields the highest phage titer is considered the optimal MOI for a particular phage-host system (37). Figure 2C shows the effect of MOI on the titer of Csp-D17. The results revealed that the optimal MOI for Csp-D17 was 0.01, with a corresponding titer of 5.5 × 10^9^ PFU/mL (*P* < 0.05). Conversely, increasing the MOI resulted in a decrease in the final phage titer, with the lowest titer (1.6 × 10^7^ PFU/mL) observed at the highest MOI tested. These findings suggest that a minimal amount of Csp-D17 (MOI = 0.01) can effectively infect the host bacterium *C. sakazakii* D17 within a micro-ecosystem. This low MOI allows for the most efficient proliferation of Csp-D17, ultimately producing the maximum number of progeny phages capable of lysing the same number of bacterial cells (38).

### One-step growth curve of phage

One-step growth curves enable the visualization of phage infectious activity throughout the replication cycle, including the latency period, burst size, and final phage titer (39). As depicted in Fig. 2D, the one-step growth curve of Csp-D17 revealed a latency period of 5 min, a burst size of 40 PFU/infected cell, and a lysis period of 60 min.

### Insensitive of BIM mutation frequencies to phage exposure

Phage infection does not universally suppress bacterial growth. Bacteria can evolve various mechanisms to resist the lytic process, giving rise to phage-insensitive mutant strains, also known as resistant strains or bacteriophage-insensitive mutants (40).

The frequency of mutations in *C. sakazakii* D17 conferring insensitivity to phage Csp-D17 was determined using the spotting method, with the resulting colonies referred to as BIMs. As shown in Table S3, *C. sakazakii* D17 yielded 31 BIM colonies following exposure to phage Csp-D17. Based on these data, the calculated frequency of *C. sakazakii* D17 mutations resulting in phage Csp-D17 insensitivity was 3.8 × 10^−7^.

### Whole-genome sequence analysis of phage

The complete genomic sequence of phage Csp-D17 has been uploaded to the NCBI GenBank database (GenBank accession number PQ656801). Analysis of the phage Csp-D17 genome, as detailed in Fig. S2 and Table S4, revealed a double-stranded DNA (dsDNA) structure with a total length of 143,081 bp. The genome exhibited a guanine + cytosine content of 46.42%. Furthermore, the genome was predicted to encode 233 open reading frames (ORFs) with an average length of 553 bp, representing approximately 90.2% of the complete genome sequence.

**Fig 3:**
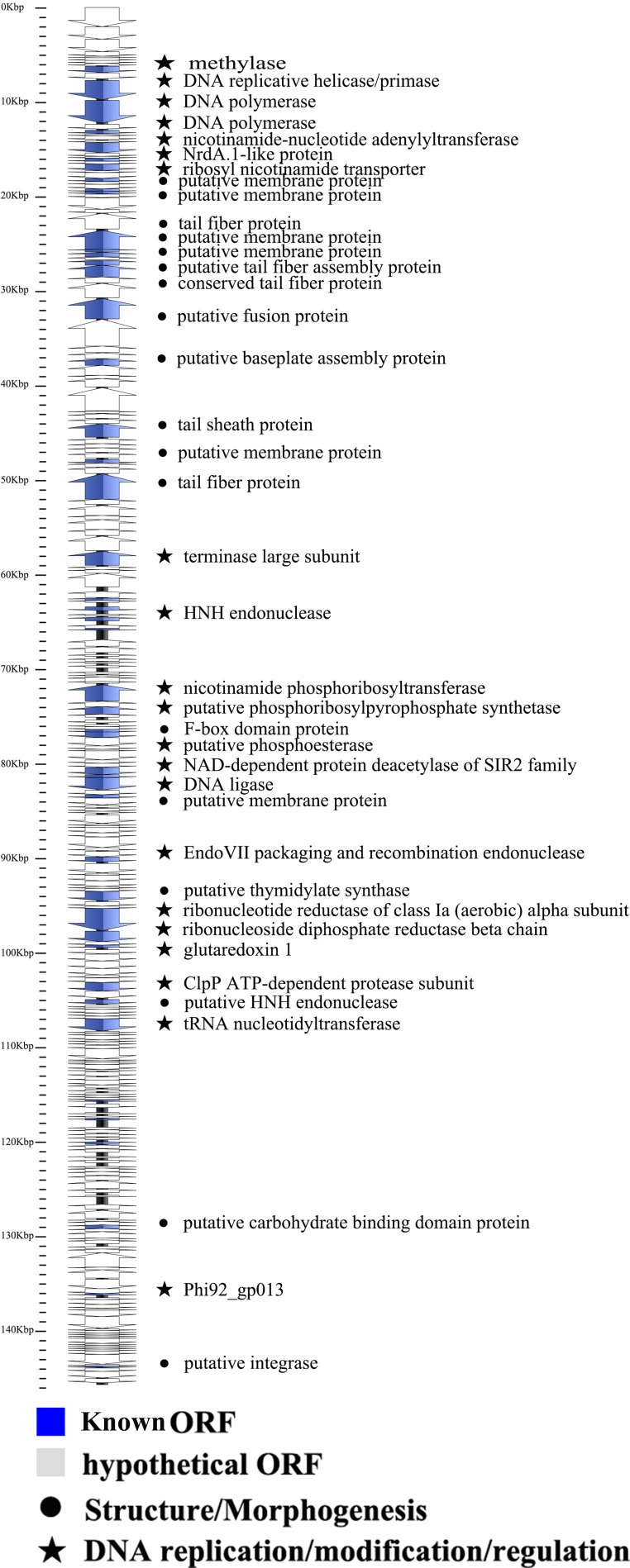
Schematic representation of the linear dsDNA genome of phage Csp-D17. An arrow with predicted functions represents the positions, orientation, and function of predicted ORFs.

Figure 3 depicts the genomic structure of phage Csp-D17, which encodes two putative tail proteins: multiple tail fiber proteins (ORF 35, ORF 39, ORF 40, ORF 63) along with a tail sheath protein (ORF 55). This finding aligns with our TEM observations that revealed a contractile tail structure. The encoded tail sheath protein facilitates phage adsorption to the host bacterium. Upon recognition of a suitable host, the sheath contracts and widens, allowing the inner tubes to reach the outer bacterial membrane. This rapid and specific recognition process precedes phage DNA injection and subsequent replication within the host (41). The Csp-D17 genome also encodes a ribosyl nicotinamide transporter (ORF 23) and several membrane proteins (ORF 26, ORF 30, ORF 36, ORF 37, ORF 51, ORF 60, ORF 111, ORF 117, ORF 118, ORF 160, ORF 171). Besides, genes encoding proteins essential for phage replication and regulation were identified, including DNA polymerase (ORF 12 and ORF 14), nicotinamide-nucleotide adenylyl transferase (ORF 18), putative thymidylate synthase (ORF 131), nicotinamide phosphoribosyl transferase (ORF 92), DNA replicative helicase/primase (ORF 11), DNA ligase (ORF 109), tRNA nucleotidyl transferase (ORF 152), and putative phosphoribosyl pyrophosphate synthetase (ORF 94). Furthermore, the genome harbors genes encoding proteins involved in thymidylate synthesis (ORF 92, ORF 11, ORF 109, ORF 152, and ORF 94). Phage-encoded lytic proteins are crucial for disrupting the host bacterial cell wall and degrading its exopolysaccharide structures, ultimately releasing progeny phages (42). Future research focusing on the functional characterization of these encoded proteins is necessary to fully exploit *C. sakazakii* phages as efficacious biocontrol agents with high lytic activity and a broad host range (43). Notably, the absence of known virulence or lysogenicity-associated genes in the Csp-D17 genome suggests its potential for safe application.

### Comparative genomic analysis

**Fig 4:**
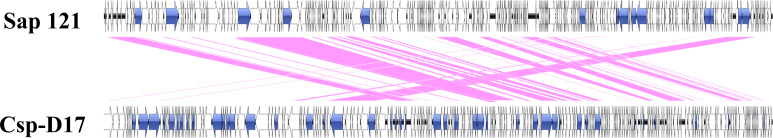
Comparative structural analyses of the phages Csp-D17 and SSE-121. Each ORF is represented by arrows indicating the size and transcriptional direction. The hypothetical proteins are shown in white, and the known ORFs are shown in blue. The pink-shaded region denotes the part of the nucleotide sequence that is more than 90% homologous.

Comparative genomic analysis of Csp-D17 and *Salmonella* phage SSE-121 (GenBank accession number JX181824) revealed a high degree of homology between the two phages (Fig. 4). A total of 90 genes shared greater than 90% sequence identity, with 85 of these encoding hypothetical proteins and the remaining 5 encoding functional proteins. The five identified functional genes encompass various essential processes within the phage life cycle. EndoVII packaging and recombination endonuclease (ORF 124) plays a role in DNA manipulation, while the terminase large subunit (ORF 70) is involved in DNA packaging. Ribonucleotide reductase of class Ia (aerobic) alpha subunit (ORF 134) contributes to the phage growth cycle. Moreover, the genome harbors genes encoding ClpP ATP-dependent protease subunit (ORF 144) and putative carbohydrate-binding domain proteins (ORF 144 and ORF 198).

### Application of phage as a biocontrol agent against *C. sakazakii* D17

**Fig 5:**
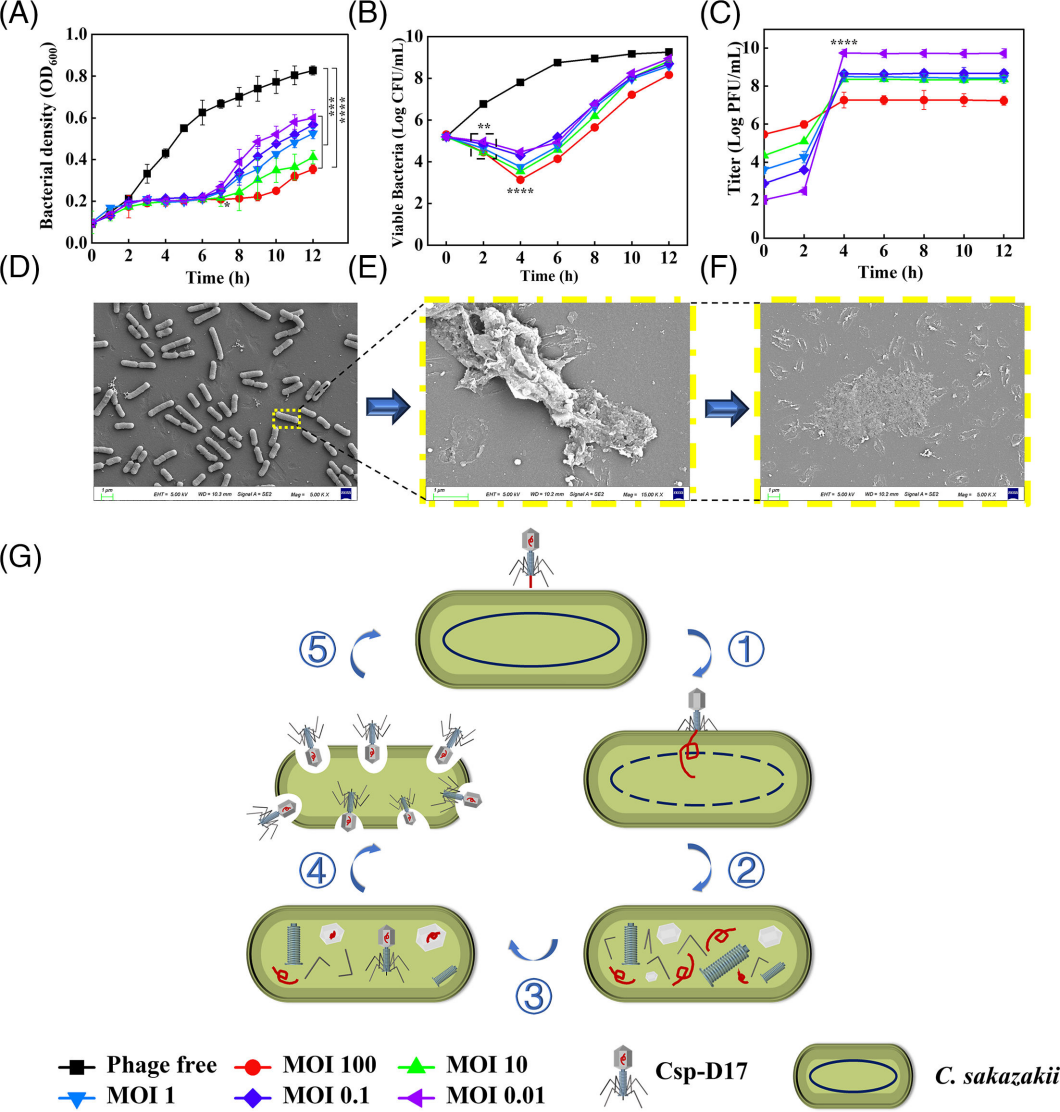
Inhibitory effect of phage Csp-D17 on *C. sakazakii* D17 in PIF at different MOIs. (**A**) Bacterial growth inhibition measured by OD_600_; (**B**) bacterial colony counts; (**C**) phage titer; (**D**) SEM micrograph of intact *C. sakazakii*; (**E and F**) SEM micrographs showing the lytic process of *C. sakazakii* D17 by phage Csp-D17 at progressive time points. Yellow boxes indicate the morphology of lysed *C. sakazakii* D17; blue arrows indicate the progressive lysis of *C. sakazakii* D17 by phage Csp-D17. (**G**) Schematic diagram illustrating the five-stage lytic cycle of phage Csp-D17: ① recognition and adsorption, ② infection, ③ replication and translation, ④ assembly, and ⑤ lysis. Scale bar: 1 µm. Significance evaluated by two-way ANOVA with Tukey’s multiple comparisons test; *P < 0.05, **P < 0.01, ***P < 0.001, ****P < 0.0001.

Csp-D17 displayed a significant inhibitory effect on *C. sakazakii* D17 (*P* < 0.001). As shown in Fig. 5A, the OD_600_ of the control group (no phage treatment) increased from 0.09 to 0.83. Conversely, all five inhibition groups with MOIs ranging from 0.01 to 100 exhibited an Imax value of 65%–67% (*P* < 0.001). This finding aligns with the established principle that adequate phage absorption onto the host bacterium’s surface can facilitate host cell lysis via endonucleases. Consequently, a positive correlation exists between phage inhibitory capacity and MOI (44). Consistently, as shown in Fig. 5A, the strongest inhibition (Imax = 67%) was observed in the group with an MOI of 100 (*P* < 0.0001). Even at the lowest MOI (0.01), Csp-D17 inhibited *C. sakazakii* D17 growth for 6 h, achieving an Imax of 65%. However, at 12 h, bacterial growth increased in both control and phage-treated groups, with the Imax in the MOI 0.01 group decreasing to 27%. This phenomenon might be attributed to the host bacterium’s restriction endonucleases, which can resist phage infection during host-phage interactions (45). The inhibitory effect of Csp-D17 on *C. sakazakii* D17 in PIF was evaluated using a simulated contamination model (Fig. 5B). The viable count of *C. sakazakii* D17 in the control group (no phage) increased from 5.20 to 9.26 log CFU/mL after incubation at 37°C for 8 h, indicating bacterial growth in PIF. In contrast, all Csp-D17-treated groups exhibited lower viable bacterial counts than the control, demonstrating the phage’s ability to inhibit *C. sakazakii* D17 growth in PIF. While the initial phase (within 2 h) of phage treatment showed an increase in viable bacterial counts for all treated groups (*P* < 0.01), the strongest inhibition was observed between 2 h and 4 h (especially at an MOI of 100, *P* < 0.0001), with viable counts ranging from 3.14 to 4.47 log CFU/mL. However, the counts started to increase again after 6 h. Notably, the final viable counts in all phage-treated groups remained lower than the control group, indicating sustained *C. sakazakii* D17 suppression in PIF. To further investigate phage dynamics, we evaluated the changes in phage titer (potency) at different MOIs within the PIF model (Fig. 5C). Csp-D17 exhibited a rapid initial proliferation for the first 2 h, followed by a sustained growth phase for the next 10 h. The highest phage titer of 10^9^ PFU/mL was observed at 12 h of incubation, with the optimal MOI being 0.01 (*P* < 0.0001). This finding corroborated our observations in the “Determination of optimal MOI” section, suggesting that within a certain threshold (no less than 10^4^ PFU/mL), a lower initial phage dose could lead to more efficient proliferation and maximize progeny phage production (46).

To elucidate the lysis mechanism, we further characterized the phage-mediated lysis process of *C. sakazakii* D17 using SEM. As shown in Fig. 5D, intact *C. sakazakii* D17 cells appeared rod-shaped with a smooth, full bacterial body and uniform size distribution. Following phage Csp-D17 addition, the phage tails attached to *C. sakazakii* D17 cells, leading to progressive damage to the bacterial structure over time (Fig. 5E). This damage manifested as wrinkling and rupturing of the bacterial cell wall. Severe disruption of cell morphology was evident, with wrinkled and ruptured cell membranes, along with bacterial aggregation and clustering. Ultimately, *C. sakazakii* D17 lost its intact morphology, with complete degradation and phagocytosis of the cellular material (Fig. 5F). This lytic process is a consequence of the biologically active phage Csp-D17 recognizing and infecting *C. sakazakii* D17. The phage then underwent a rapid replication cycle encompassing five distinct phases: adsorption (recognition), invasion, proliferation (including protein synthesis and genome replication), assembly, and release (Fig. 5G). Bioinformatic analysis of the Csp-D17 genome revealed the presence of four tail fiber protein genes (ORF 35, ORF 39, ORF 40, and ORF 63; see Table S5 for details). These genes encode proteins that recognize and infect host bacteria through specific receptor-binding proteins (RBPs). This recognition and infection mechanism enables Csp-D17 to target and lyse its host. In conclusion, Csp-D17 employs its unique tail proteins to recognize, infect, and lyse *C. sakazakii* D17. This targeted action effectively reduces *C. sakazakii* D17 viability, inhibiting bacterial growth and promoting phage Csp-D17 proliferation (Fig. 5G).

## DISCUSSION

*C. sakazakii* infections significantly threaten global public health and safety (6). Phages, with their ability to specifically target and lyse bacteria while producing new progeny phages, offer a promising new avenue for the bioprevention and control of *C. sakazakii* contamination in food and food production environments (47).

Phages offer a promising approach for the biological control of *C. sakazakii* due to their ubiquity in the environment, ease of production, and cost-effectiveness (48). One *C. sakazakii* phage strain, Csp-D17, was isolated and characterized from environmental wastewater samples. Interestingly, the plaque diameter of Csp-D17 differed significantly from that of EspYZU13 (4.81 mm) reported in previous studies (19). However, TEM revealed that both phages possess contractile tail sheaths and belong to the order *Caudoviricetes* within the *Ackermannviridae* family. This observation contrasts with the tail structures of *C. sakazakii* phages from the *Autographiviridae* and *Ackermannviridae* families described elsewhere (49). These findings highlight the remarkable morphological and genetic diversity among C. *sakazakii* phages. Furthermore, compared to the lytic spectra of previously documented bacteriophages, Esp-YZU14 (50% efficacy, 5/10 strains) (19) and LPCS7 (20%, *n* = 2/10 strains) (50) against *C. sakazakii*, phage Csp-D17 demonstrated intermediate activity (36.4%, *n* = 4/11 strains). Accordingly, Csp-D17 could lyse *C. sakazakii* strains in real-world complex environments when administered in a concentrated or cocktail formulation as opposed to the *Cronobacter* phage JK004 (*n* = 1/11 strains) (15). Such properties position *C. sakazakii* as a promising candidate for targeted biocontrol applications within PIF manufacturing environments. Phage stability under various environmental conditions, particularly temperature and pH, is crucial for their efficacy in controlling foodborne pathogens (51). Csp-D17 exhibited remarkable tolerance to temperature (50°C–70°C) and pH (6–9). This stability was broader than other phages, such as JK004 (30°C–40°C, pH 6–8) (15). The wider tolerance range suggests that Csp-D17 may be applicable in various real-world food processing environments. Studies have reported that the optimal MOIs for *C. sakazakii* phages LPCS28 (50) and A24 (52) were 1 and 0.1, respectively. In contrast, Csp-D17 displayed a significantly lower optimal MOI (0.01). This translated to a lower initial phage inoculum (no less than 10^4^ PFU/mL) required to achieve a measurable effect on the host bacteria. This characteristic facilitates large-scale phage production and application in practical settings. Csp-D17 demonstrated a shorter incubation period (5 min) compared to other *C. sakazakii* phages like EspYZU15 (20 min) (19), EspYZU08 (40 min) (49), and ΦCS01 (60 min) (53). Besides, it exhibited a larger lysis volume (40 PFU/cell) compared to EspYZU13 (10 PFU/cell) (19). Furthermore, Csp-D17 displayed a lower frequency of emergence of insensitive mutations in *C. sakazakii* D17 compared to phages SYZU08 (BIM = 5.4 × 10^−6^) and SYZU09 (BIM = 5.3 × 10^−6^) reported by Yang et al. (54) for *Shewanella baltica*. This characteristic strengthens the potential of Csp-D17 as a biocontrol agent. As previously established, phage lysogenicity is a crucial safety factor (24). Notably, Csp-D17 did not exhibit lysogenic properties. However, Li et al. (23) reported that phage vB_VcaS_HC, despite displaying strong lytic activity and no evidence of lysogenic conversion, harbored a lysogen-associated recombinase gene (*recA*) upon whole-genome sequencing. This finding highlights the importance of whole-genome sequencing, gene annotation, and comparative genome analysis for ensuring phage safety and efficacy before biocontrol applications (55). Whole-genome sequencing revealed that Csp-D17 possesses a novel, dsDNA genome structure with overlapping cluster ends. This architecture allows for circular genome replication, a feature observed in other phages with similar terminal structures (45). Moreover, TEM confirmed that Csp-D17 belongs to the *Ackermannviridae* family. However, its genome exhibited significant divergence from that of EspYZU13 (19), highlighting the remarkable genetic diversity within *C. sakazakii* phages. In addition, a BLASTP search of GenBank for Csp-D17 revealed low homology (84.24% with 67% coverage) with *Cronobacter* phage vB_CsaM_GAP31 genomes. This finding suggests that phage Csp-D17 is a novel lytic *Cronobacter* phage. Interestingly, Csp-D17 exhibited a high homology to *Salmonella* phage SSE-121, isolated from Baltimore harbor water in 2012, consistent with findings reported by Zhou et al. (56), indicating that phages may share some ORFs. Co-linearity analysis further elucidated the relationship between Csp-D17 and *Salmonella* phage SSE-121. It was found that phage Csp-D17 and *Salmonella* phage SSE-121 exhibited homology in specific gene regions. However, this homology was not uniformly present across the entire genome, appearing predominantly in hypothetical proteins and a few function-related gene modules. Similar to findings reported by Zhou et al. (31), similar domains or conserved sequences were identified in DNA packaging (terminase large subunit) and manipulation-related genes (EndoVII packaging and recombination endonuclease). These shared elements may be related to their coordinated roles in phage replication, assembly, and host manipulation, although the extent of homology and functional connections warrants further study. While genomic homology suggests evolutionary relationships between phages, it does not necessarily confer functional equivalence. In the present study, only 74.92% sequence similarity was observed in host recognition and infection-related genes (particularly tail fiber proteins) between the phages, which likely accounts for their divergent host ranges and infection efficiencies. This genomic divergence may account for the limited ability of phage Csp-D17 to lyse *Salmonella* (CICC 21513), potentially due to distinct host recognition mechanisms and variations in lytic receptor specificity. Besides, the bacteriophage Csp-D17 (excluding its host bacterium) exhibited lytic activity against the other three strains of *C. sakazakii* of different origins tested (CICC 22919, BQW100020180011, and BQW100020180013) in this experiment. Interestingly, phage Csp-D17 interacts with its host receptor via the RBP located in its tail, thereby initiating the adsorption, infection, and lysis processes. Overall, the receptors of the aforementioned three strains of *C. sakazakii* may functionally mimic those of its host during Csp-D17-mediated lysis. Recent findings by Ding et al. (57) support this view, suggesting that the interaction between phage RBPs and their receptors determines the specificity of the host range. The efficiency of plaque can be used in follow-up studies to evaluate the infectivity of phages in different strains of *C. sakazakii*. Besides those, the genome size of Csp-D17 (233 functional genes) is reportedly larger than that of phage JK004 (123 functional genes) (15). This difference can be attributed to the varying number of genes encoding functionally related proteins. Similarly, the genomes of phage CBT2 (179,308 bp) (48) and A24 (75,106 bp) (52) differ in size. The larger CBT2 genome, encoding 279 functional proteins, is 104,202 bp longer than the A24 genome, which harbors only 108 ORFs. Tailed phages, as established in previous studies (58–61), utilize tail fibers to recognize and bind to specific bacterial receptors during the adsorption process. The diversity of tail fibers enables phages to efficiently recognize and infect a broader range of bacterial hosts (62, 63). In this study, Csp-D17 encoded various tail fiber proteins (ORF 35, ORF 39, ORF 40, and ORF 63) potentially involved in bacterial receptor recognition. This characteristic might contribute to Csp-D17’s ability to recognize, infect, and lyse different *C. sakazakii* species. In contrast, the phage EspYZU05, characterized by Li et al. (49), encoded tail-associated proteins within only three open reading frames (ORFs 35, 36, and 40). Transmission electron microscopy revealed that EspYZU05 possessed an ortho-polyhedral head structure, approximately 55 nm in diameter, and a tail length of approximately 18 nm. These morphological characteristics aligned with those of the *Caudoviricetes* order and the *Ackermannviridae* family. The observed morphological disparity between the two phages may be attributed to variations in the number of encoded tail proteins. Furthermore, the absence of virulence and lysogeny-associated genes within the Csp-D17 genome enhances its safety for biocontrol applications in the food production industry.

This study evaluated the inhibitory effect of phage Csp-D17 on *C. sakazakii* D17 in artificially contaminated PIF at various MOIs (100, 10, 1, 0.1, 0.01). Csp-D17 demonstrated inhibitory activity against *C. sakazakii*, suggesting its potential applicability to a broad range of PIF brands and potentially other dairy products. Previous studies have explored using other *C. sakazakii* phages for inhibition, but these usually required high phage titers. For instance, phages LPCS28 (50) (MOI of 10^3^–10^4^), A24 (52) (MOI of 10^5^), CBT2 (48) (MOI of 10^7^–10^2^), and JK004 (15) (MOI of 10^4^) exhibited limited bacterial control at lower titers in PIF samples. In contrast, phage Csp-D17 displayed effective *C. sakazakii* D17 control at a low MOI (10^2^), highlighting its potential inhibitory potential. Furthermore, real-time monitoring of the inhibition process using SEM provided a vivid and intuitive representation of the lysed host bacteria. This approach corroborated the inhibitory efficacy of Csp-D17 and visually confirmed its ability to specifically recognize, infect, and lyse *C. sakazakii* D17 through its unique tail proteins, as previously established (64, 65).

In conclusion, this study employed biological characterization, genomic analysis, and practical application to evaluate phage Csp-D17. Our results suggest that Csp-D17 functions as a safe biological inhibitor for *C. sakazakii*, characterized by its rapid replication cycle encompassing assembly and lysis during host infection.

### Conclusion

This study successfully isolated a bacteriophage strain exhibiting underlying lytic activity against foodborne *C. sakazakii*. The biological properties, complete genome sequence, and potential application of this phage for *C. sakazakii* D17 biocontrol in PIF were comprehensively characterized. Our findings demonstrate the promise of this phage as a novel biocontrol agent for mitigating *C. sakazakii* contamination in dairy products. However, in practical applications such as in PIF production facilities, host bacteria can readily develop resistance to individual phages. Therefore, employing a mixture of different phages (phage cocktail) can extend the host range of phage preparations and reduce the mutation frequency of host bacteria to phages. In addition, the lysis rate test revealed that Csp-D17 had a low lysis efficiency. Therefore, to better combat future PIF contamination by *C. sakazakii*, the isolation of additional phages with a wider host range and enhanced efficiency is imperative.

### Highlights

- Csp-D17 was a novel lytic phage against *C. sakazakii* isolated from environmental wastewater.
- Csp-D17 was subjected to encompassing morphological, physiological, and whole-genomic analyses.
- Csp-D17 can be acted as a potential novel biocontrol agent to control *C. sakazakii* in PIF.

## Data Availability

The complete genomic sequence of phage Csp-D17 has been uploaded to the NCBI GenBank database (GenBank accession number PQ656801).

## SUPPLEMENTAL MATERIAL

Supplemental materials spectrum.00731-25-s0001.docx Supplemental method, Figures S1 and S2, and Tables S1 to S5.

ASM does not own the copyrights to Supplemental Material that may be linked to, or accessed through, an article. The authors have granted ASM a non-exclusive, world-wide license to publish the Supplemental Material files. Please contact the corresponding author directly for reuse.
