# Supplementary material for: Characterization, genomics, and applications of the Cronobacter sakazakii bacteriophage Csp-D17
Source: Microbiol Spectr. 2025 Nov 11;13(12):e00731-25. doi: 10.1128/spectrum.00731-25 (PMC12671138; doi:10.1128/spectrum.00731-25)
Supplement: Supplemental materials — Supplemental method, Figures S1 and S2, and Tables S1 to S5. [file spectrum.00731-25-s0001.docx]

Supporting Information for

**Characterization, Genomics, and Applications** **of the *Cronobacter sakazakii* Bacteriophage Csp-D17**

Yue-yue Zhang ^1^, Ding-rong Zhang ^2^, Zhen-quan Yang ^3^, Lei Yuan ^3^, Ya Zhao ^1, *^, Yuan-song Zhang ^4, **^

^a^ *Department of Cardiology, Shanghai Yangpu District Kongjiang Hospital, Shanghai 200093, China*

^b^ *Department of Neurosurgery, Chongqing Municipal Corps Hospital of Chinese People’s Armed Police Force, Chongqing 400061, China*

^c^ *School of Food Science and Engineering, Yangzhou University, Yangzhou, Jiangsu 225127, China*

^d^ *Department of Nutrition, Shanghai East Hospital, Tongji University School of Medicine, Shanghai, 200120, China*

*Corresponding author: Shanghai Yangpu District Kongjiang Hospital, No.480, Shuangyang Road, Shanghai, 200093, China

**Corresponding author: Shanghai East Hospital, Tongji University School of Medicine, No. 150, Jimo Road, Pudong, Shanghai 200120, China.

E-mail address: [Zhangys695667422@163.com](mailto:Zhangys695667422@163.com) (Yuan-song Zhang).

Yue-yue Zhang and Ding-rong Zhang contributed equally to this article. Author order was determined  in order of increasing seniority.

1. Methods

1.1 Isolation, purification, and concentration of *C. sakazakii* phage

Based on the method described by Chen et al. (2022), 32 sewage samples were resuspended by centrifugation (DICO-220, Thermo Fisher Scientific Corporation, MA, USA) at 8,000 rpm for 10 min. The supernatant was then collected and decontaminated by filtration through a 0.22 µm filter membrane. 5 mL of the filtrate and 100 µL of the host bacteria were separately added to 5 mL of 2 × NB liquid medium. The mixture was thoroughly mixed and incubated overnight at 37℃. Subsequently, the culture medium was centrifuged at 8,000 rpm for 10 min at 4℃. The supernatant was then decontaminated by filtration through a 0.22 µm membrane filter to obtain a phage-enriched solution. This solution was stored at 4℃ for a short-term period. For long-term storage, the remaining phage-enriched solution was aliquoted (divided into portions). 600 μL of the solution was mixed with 300 µL of sterile 70% glycerol in a sterile freezing tube. The mixture was thoroughly vortexed and stored at -80℃.

Next, following the method of Yang et al. (2023), 200 µL of phage enrichment solution and 200 µL of host bacteria D17 were combined. 10 mL of NB soft agar (0.7% agar) was added, and the mixture was thoroughly mixed. The mixture was then poured onto NB plates containing 1.5% agar. After allowing the top layer to solidify for 5 min, the plates were incubated at 37℃ for 8 h. Phage plaques were picked from the solidified agar and transferred to 900 µL of SM buffer (8 mM MgSO_4_∙7H_2_O, 100 mM NaCl, 5 mL/L of 2% gelatin solution, and 50 mM Tris-HCl, pH 7.5). The suspension was vortexed thoroughly to ensure complete homogenization. The supernatant was collected and subjected to serial dilutions. The plaque formation assay was repeated 3 - 5 times for phage purification. To determine phage titer using the double-layer agar method, 100 µL of a phage dilution was mixed with 100 µL of host bacteria culture (adjusted to a concentration of 10^9^ CFU/mL). This mixture was then added to 5 mL of NB soft agar (0.7% agar) and poured onto an NB plate containing 1.5% agar. The top layer was allowed to solidify for 5 min, and the plate was incubated at 37℃ for 8 h. Three replicate plates were prepared for each dilution. Following incubation, the number of phage plaques formed on each plate was counted. The formula for calculating phage titer (plaque-forming units per milliliter, PFU/mL) is shown in Eq. (1).

$\text{Titer= }\frac{\overline{\text{x}}}{\text{0.1}\text{×d}}$ (1)

Where, titer is the phage potency (PFU/mL), $\overline{\text{x}}$ is the average phage vacuole number (PFU), 0.1 is the inoculum volume (mL), and d is the sample dilution.

Phage concentration was performed with slight modifications based on the method described by Tang et al. (2023b). Briefly, DNase I (final concentration of 1 μg/mL; Sangon Bioengineering Co., Ltd., Shanghai, China) and RNase A (final concentration of 1 μg/mL; Sangon Bioengineering Co., Ltd., Shanghai, China) were added to the phage amplification solution and mixed thoroughly. Subsequently, NaCl (final concentration of 1 M; Sinopharm Chemical Reagent Co., Ltd., Beijing, China) was added, followed by thorough mixing. The mixture was then incubated on ice for 2 h. The precipitate was removed by centrifugation (8000 *g*, 10 min, 4℃), and the supernatant was collected. Next, polyethylene glycol (PEG) 8000 (10% w/v; Sangon Bioengineering Co., Ltd., Shanghai, China) was added to the supernatant, and the mixture was incubated on ice for an additional 5 h. The supernatant was again discarded by centrifugation (8000 *g*, 10 min, 4℃). Finally, the phage pellet was resuspended in 1 mL of SM buffer. This constituted the concentrated phage solution. For further purification, a cesium chloride (CsCl) density gradient was employed. Briefly, 3 mL each of 1.7 g/mL, 1.5 g/mL, and 1.45 g/mL CsCl solutions (Sanger Biotech Co., Ltd., Shanghai, China) were sequentially layered in ultracentrifuge tubes. Two milliliters of the concentrated phage solution were then loaded onto the gradient. The tubes were centrifuged at 30,000 rpm for 2 h. The phage-containing band between the 1.5 g/mL and 1.45 g/mL CsCl layers was aspirated and stored at 4℃.

1.2 Determination of the frequency of insensitive mutation

The frequency of bacteriophage-insensitive mutants (BIMs) in the *C.* *sakazakii* D17 was determined using a method described by Gutiérrez et al. (2015), with slight modifications. The initial step involved isolating putative BIM strains. Briefly, 100 μL of the bacteriophage solution (at a concentration of 10^8^ PFU/mL) was thoroughly mixed with 100 μL of the host bacterial culture (adjusted to a concentration of 10^6^ CFU/mL). In the experimental group, 50 μL of CaCl_2_ (10 mmol/L) and 50 μL of MgSO_4_ (10 mmol/L) were added to the mixture to induce BIM formation. The control group received 100 μL of 0.9% sterile saline solution instead of the CaCl_2_ and MgSO_4_ solution. Following a 10-min incubation at 37℃, 100 μL aliquots from both the experimental and control groups were plated onto separate NB agar plates. The plates were then incubated at 37℃ for 18 h. Colonies that grew on the experimental group plate but not on the control group plate were presumptively identified as BIMs of *C. sakazakii* D17.

Following the isolation step, the putative BIMs were further evaluated for phage insensitivity using a spot test method. Each putative BIM isolate was inoculated into a separate tube containing 5 mL of NB liquid medium and incubated at 37℃ for 12 h. Subsequently, a 2 μL aliquot of the bacterial culture was streaked three to four times for single-colony isolation onto an NB agar plate. After allowing the streaks to dry, a single, well-isolated colony was selected and inoculated into another 5 mL of fresh NB liquid medium. The culture was again incubated at 37℃ for 12 h. To assess phage sensitivity, 10 μL of the resulting bacterial suspension was pipetted onto a separate NB agar plate and allowed to settle and dry completely. Then, 2 μL of the bacteriophage solution (at a concentration of 10^9^ PFU/mL) was spotted in the same area where the bacteria were previously deposited. The plate was again allowed to settle and dry before incubation at 37℃ for 8 h. Following incubation, the plate was examined for a clear zone of lysis surrounding the area where the phage was spotted. The absence of a clear zone indicated a lack of phage sensitivity, and the bacterial isolate was then confirmed as a BIM.

The formula for calculating the frequency of insensitive mutations is shown in Eq. (4).

*BIM = N/10^6^* (4)

Where, BIM is the frequency of insensitive mutations, N is the identified insensitive mutant strain, and 10^6^ is the host bacterial concentration (CFU/mL).

**Figure S1**

**
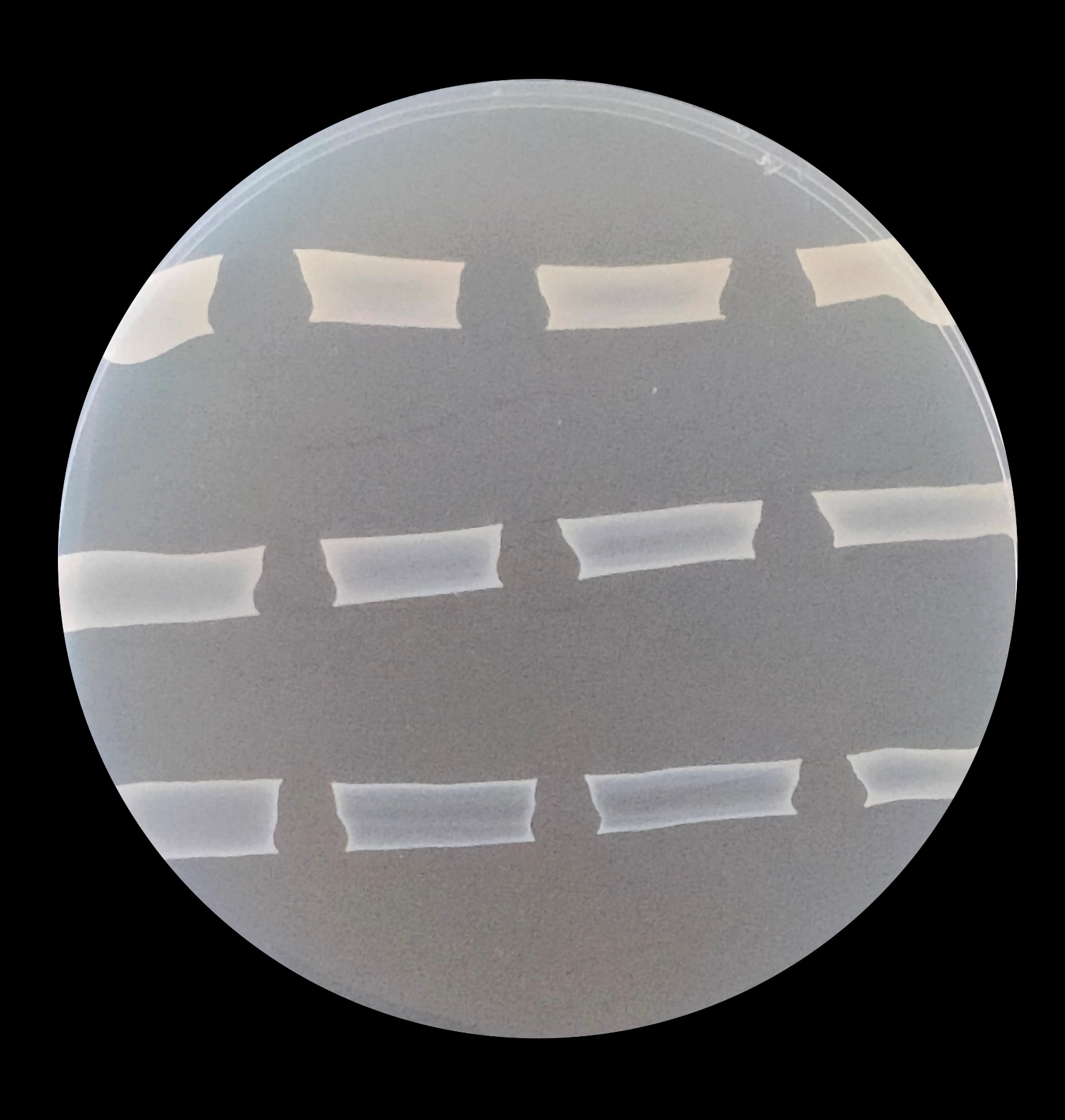
**

**Fig.S1.** Lysogeny test of phage Csp-D17. The yellow box represents the absence of bacterial growth at the intersection of phage Csp-D17 and *C. sakazakii* D17 in the spot test, confirming the non-lysogenic nature of the phage.

**Figure S2**


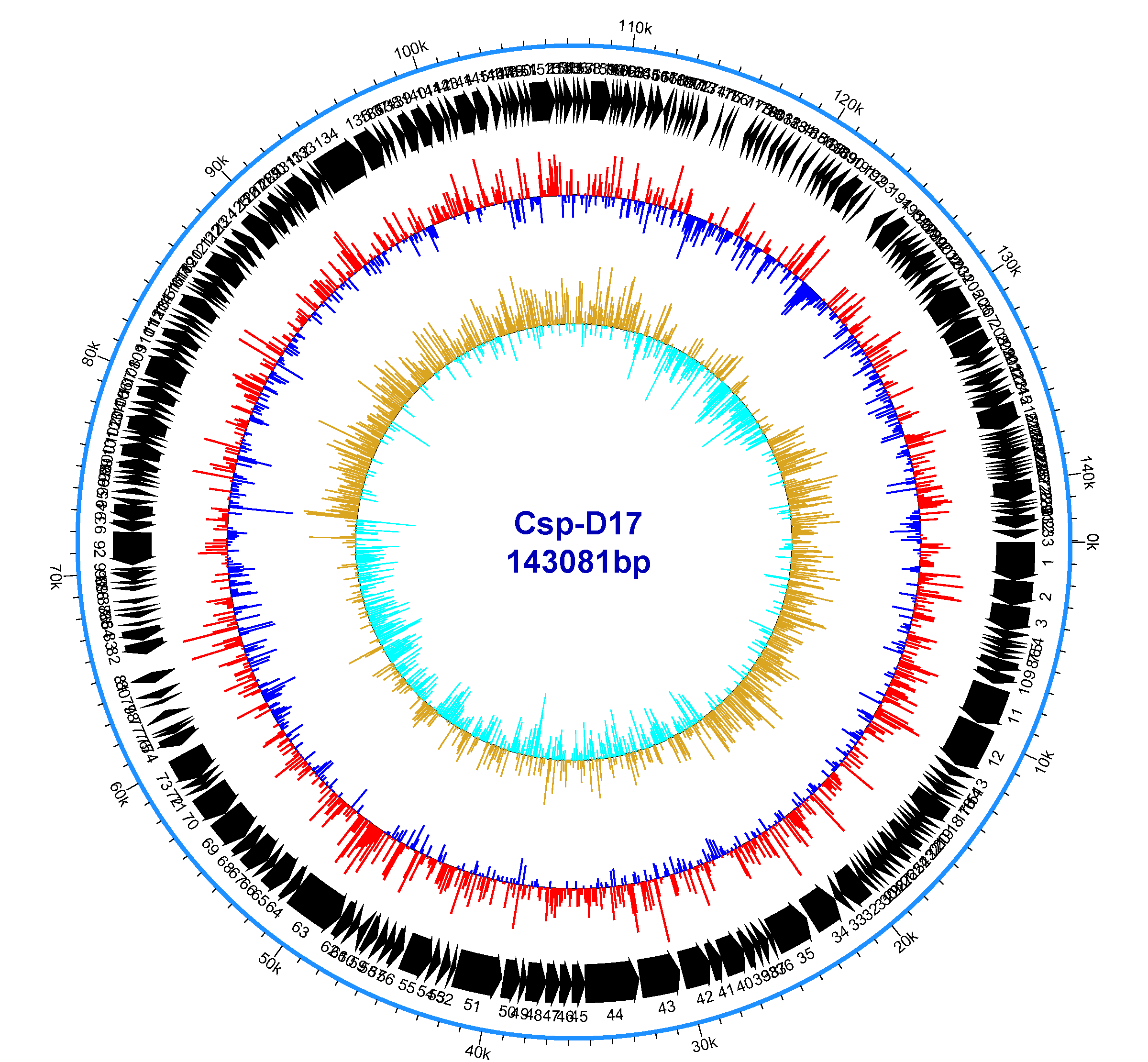


**Fig. S2.** Genomes circles of Csp-17. From the outside, circles display (1) Open reading frames (ORFs) transcribed clockwise or counterclockwise. (2) G + C % content. (3) GC skew: values greater than zero are in magenta, and smaller values are in green. (4) Physical map scaled in Kbp.

**Table S1** All strains used in this study.

| **No.** | **Species** | **Strain No.** | **Stored** | **Source** | **Ref.** |
| --- | --- | --- | --- | --- | --- |
| 1 | *C. sakazakii* | CICC 21560 | China Center of Industrial Culture Collection (CICC) | Unknown | Li et al. (2021) |
| 2 |  | CICC 22919 |  | Unknown |  |
| 3 |  | CICC 21545 |  | Milk Powder |  |
| 4 |  | CICC 21673 |  | Unknown |  |
| 5 |  | CICC 21569 |  | Chocolate-chip Biscuit |  |
| 6 |  | BQW100020180009 | Yang Zhou Public Health Center | Milk Powder | Zhang et al. (2023) |
| 7 |  | BQW100020180010 |  |  |  |
| 8 |  | BQW100020180011 |  |  |  |
| 9 |  | BQW100020180012 |  | Clinical Sample |  |
| 10 |  | BQW100020180013 |  |  |  |
| 11 | *E. coli* | CICC 10664 | China Center of Industrial Culture Collection (CICC) | Standard strain |  |
| 12 | *S.* Enteritidis | CICC 21513 |  | Frozen Mizuhopecten yessoensis |  |
| 13 | *S. aureus* | CICC 21600 |  | Unknown |  |
| 14 | *B. cereus* | CICC 21261 |  | Unknown |  |
| 15 | *L. monocytogenes* | ATCC 19111 | American Type Culture Collection (ATCC) | Poultry |  |
| 16 | *E. hormaechei* | YZU2-5 | Food Quality and Safety Laboratory in Yangzhou University | Pork | Chen et al. (2022) |
| 17 | *V. parahaemolyticus* | VpYZU84 |  | Bass | Tang et al. (2023b) |
| 18 | *B. thermosphacta* | YZU-2-7 |  | Spoiled meat | Tang et al. (2023a) |
| 19 | *C. sakazakii* | D17 |  | Soymilk | This work |

**Table S2** The lysis spectrum of Csp-D17.

| **No.** | **Species** | **Strain No.** | **Csp-D17** | **Lytic Activity ^#^ (%)** |
| --- | --- | --- | --- | --- |
| 1 | *C. sakazakii* | CICC 21560 | - | 36.4%  (4/11) |
| 2 |  | CICC 22919 | + |  |
| 3 |  | CICC 21545 | - |  |
| 4 |  | CICC 21673 | - |  |
| 5 |  | CICC 21569 | - |  |
| 6 |  | BQW100020180009 | - |  |
| 7 |  | BQW100020180010 | - |  |
| 8 |  | BQW100020180011 | + |  |
| 9 |  | BQW100020180012 | - |  |
| 10 |  | BQW100020180013 | + |  |
| 11 | *E. coli* | CICC 10664 | - |  |
| 12 | *S.* Enteritidis | CICC 21513 | - |  |
| 13 | *S. aureus* | CICC 21600 | - |  |
| 14 | *B. cereus* | CICC 21261 | - |  |
| 15 | *L. monocytogenes* | ATCC 19111 | - |  |
| 16 | *E. hormaechei* | YZU2-5 | - |  |
| 17 | *V. parahaemolyticus* | VpYZU84 | - |  |
| 18 | *B. thermosphacta* | YZU-2-7 | - |  |
| 19 | *C. sakazakii* | D17 | Host |  |

**^#^**: Only the lysis rate in *C. sakazakii*; +: plaque formation; -: no plaque formation

**Table S3** Frequency of insensitive mutations in Csp-D17.

| Phage | Putative number of BIM colonies (CFU) | Number of BIM colonies (CFU) | Initial number of bacteria (CFU) | Mutation frequency |
| --- | --- | --- | --- | --- |
| Csp-D17 | 66 | 31 | 8.1 × 10^7^ | 3.8 × 10^-7^ |

**Table S4** Statistical representation of predicted results of coding genes.

| Sample ID | Genome size (bp) | GC Content (%) | Gene number | Gene total length (bp) | Gene average length (bp) | Gene length / Genome (%) |
| --- | --- | --- | --- | --- | --- | --- |
| Csp-D17 | 143081 | 46.42 | 233 | 129048 | 553 | 90.2 |

**Table S5** Genomic function annotation of phage Csp-D17.

| **ORFs** | **Start** | **End** | **Strand** | **Length** | **Molecular mass** | **pi** | **Putative function** | **Best phage homolog (identities %)** | **Accession** | **Best evalue** |
| --- | --- | --- | --- | --- | --- | --- | --- | --- | --- | --- |
| 1 | 1 | 1995 | + | 664 | 76006.1 | 6.74 | hypothetical protein | *Salmonella* phage SSE121  98.6 | AFU63727.1 | 0.00E+00 |
| 2 | 1992 | 3278 | + | 428 | 47136.1 | 4.93 | hypothetical protein | *Salmonella* phage SSE121  95.1 | AFU63726.1 | 7.20E-234 |
| 3 | 3278 | 4582 | + | 434 | 48570.7 | 8.09 | hypothetical protein | *Salmonella* phage SSE121  92.6 | AFU63725.1 | 7.10E-237 |
| 4 | 4593 | 5042 | + | 149 | 17216.6 | 10.04 | hypothetical protein GAP31_264 | *Cronobacter* phage vB_CsaM_GAP31  68.9 | AFC21445.1 | 7.20E-56 |
| 5 | 5044 | 5223 | + | 59 | 6923.7 | 4.47 | hypothetical protein BIS47_246 | *Klebsiella* phage vB_KpnM_BIS47  74.6 | ARB12750.1 | 4.20E-15 |
| 6 | 5204 | 5518 | + | 104 | 11626 | 3.93 | hypothetical protein GAP31_262 | *Cronobacter* phage vB_CsaM_GAP31  77.1 | AFC21443.1 | 1.50E-36 |
| 7 | 5511 | 5795 | + | 94 | 11383.3 | 4.07 | hyphothetical protein | *Escherichia* phage 4MG  79.5 | AGZ17482.1 | 8.10E-37 |
| 8 | 5795 | 6058 | + | 87 | 9570.7 | 4.43 | hypothetical protein SP21_101 | *Salmonella* phage 21  95.9 | AKJ74488.1 | 1.30E-33 |
| 9 | 6111 | 6761 | + | 216 | 24761.1 | 8.68 | methylase | *Salmonella* phage 21  99.1 | AKJ74489.1 | 1.20E-133 |
| 10 | 6784 | 7407 | + | 207 | 23045.8 | 9.26 | hypothetical protein OMEGA_68 | *Klebsiella* phage vB_KpnM_KaOmega  83.1 | QEG12136.1 | 2.40E-94 |
| 11 | 7575 | 9587 | + | 670 | 74962.8 | 5.44 | DNA replicative helicase/primase | *Cronobacter* phage vB_CsaM_GAP31  96.9 | AFC21438.1 | 0.00E+00 |
| 12 | 9650 | 12022 | + | 790 | 91091.2 | 8.13 | DNA polymerase | *Salmonella* phage PVPSE1  99.5 | ADP02411.1 | 0.00E+00 |
| 13 | 12145 | 12636 | + | 163 | 18602.9 | 9.27 | hypothetical protein | *Salmonella* phage SSE121  98.8 | AFU63711.1 | 2.60E-91 |
| 14 | 12737 | 13120 | + | 127 | 14460.5 | 5.29 | DNA polymerase | *Salmonella* phage 40  89.9 | AKJ73498.1 | 1.30E-58 |
| 15 | 13155 | 13352 | + | 65 | 7246.3 | 4.44 | hypothetical protein | *Salmonella* phage SSE121  96.9 | AFU63708.1 | 2.80E-28 |
| 16 | 13354 | 13752 | + | 132 | 14939.8 | 5.8 | hypothetical protein GAP31_253 | *Cronobacter* phage vB_CsaM_GAP31  84.1 | AFC21434.1 | 4.00E-58 |
| 17 | 13826 | 14032 | + | 68 | 7832.2 | 8.5 | hypothetical protein SP40_53 | *Salmonella* phage 40  66.2 | AKJ73494.1 | 1.00E-20 |
| 18 | 14044 | 15117 | + | 357 | 41149.6 | 6.33 | nicotinamide-nucleotide adenylyltransferase | *Salmonella* phage PVPSE1  91.9 | ADP02421.1 | 5.70E-192 |
| 19 | 15119 | 15484 | + | 121 | 13886.2 | 4.31 | hyphothetical protein | *Escherichia* phage 4MG  93.3 | AGZ17494.1 | 2.70E-61 |
| 20 | 15471 | 15674 | + | 67 | 7860.8 | 4.44 | hypothetical protein GAP31_247 | *Cronobacter* phage vB_CsaM_GAP31  75.4 | AFC21428.1 | 7.30E-24 |
| 21 | 15674 | 16003 | + | 109 | 12416.4 | 8.64 | NrdA.1-like protein | *Escherichia* phage 4MG  75 | AGZ17496.1 | 2.10E-44 |
| 22 | 15996 | 16253 | + | 85 | 9446.5 | 4.08 | hypothetical protein CPT_Mydo_270 | *Proteus*  phage Mydo  85.9 | AZF87822.1 | 1.50E-34 |
| 23 | 16255 | 16971 | + | 238 | 26796.5 | 9.52 | ribosyl nicotinamide transporter | *Klebsiella* phage vB_KpnM_KB57  93.7 | ALM02423.1 | 1.70E-123 |
| 24 | 16968 | 17150 | + | 60 | 6289.6 | 8.04 | hypothetical protein KB57_032 | *Klebsiella* phage vB_KpnM_KB57  50 | ALM02425.1 | 4.50E-09 |
| 25 | 17160 | 17729 | + | 189 | 20943.3 | 9.77 | hyphothetical protein | *Escherichia* phage 4MG  88.4 | AGZ17503.1 | 1.10E-93 |
| 26 | 17733 | 18104 | + | 123 | 14242.6 | 7.5 | putative membrane protein | *Cronobacter* phage vB_CsaM_GAP31  80.5 | AFC21421.1 | 3.20E-57 |
| 27 | 18095 | 18373 | + | 92 | 10808.3 | 4.47 | hypothetical protein | *Salmonella* phage SSE121  92.4 | AFU63695.1 | 1.80E-44 |
| 28 | 18366 | 18860 | + | 164 | 17477 | 8.7 | hypothetical protein | *Salmonella* phage SSE121  65.9 | AFU63694.1 | 3.40E-22 |
| 29 | 18857 | 19123 | + | 88 | 9478.7 | 4.94 | / | / | / | 7.60E-14 |
| 30 | 19120 | 19347 | + | 75 | 8307.3 | 11.41 | putative membrane protein | *Raoultella* phage Ro1  62.5 | AUE23438.1 | 2.00E-53 |
| 31 | 19407 | 19754 | + | 115 | 12712.1 | 4.08 | hypothetical protein GAP31_234 | *Cronobacter* phage vB_CsaM_GAP31  92.2 | AFC21415.1 | 4.50E-182 |
| 32 | 19768 | 20952 | + | 394 | 41787.7 | 5.3 | hypothetical protein | *Salmonella* phage SSE121  84.1 | AFU63690.1 | 2.10E-30 |
| 33 | 20969 | 21367 | + | 132 | 15330.3 | 10.08 | hypothetical protein Ro1_00230 | *Raoultella* phage Ro1  49.6 | AUE23435.1 | 2.70E-222 |
| 34 | 21400 | 22992 | - | 530 | 55396.6 | 3.95 | hypothetical protein GAP31_232 | *Cronobacter* phage vB_CsaM_GAP31  74.5 | AFC21413.1 | 4.40E-198 |
| 35 | 23158 | 25125 | - | 655 | 66657.1 | 3.99 | tail fiber protein | *Salmonella* phage SSE121  66.6 | AFU63688.1 | 3.70E-41 |
| 36 | 25145 | 25399 | - | 84 | 9654.2 | 9.93 | putative membrane protein | *Cronobacter* phage vB_CsaM_GAP31  95.2 | AFC21411.1 | 3.20E-70 |
| 37 | 25430 | 25903 | - | 157 | 18016.8 | 8.32 | putative membrane protein | *Cronobacter* phage vB_CsaM_GAP31  88.5 | AFC21410.1 | 1.10E-34 |
| 38 | 25900 | 26214 | - | 104 | 12232.2 | 6.95 | hypothetical protein | *Salmonella* phage SSE121  94.2 | AFU63685.1 | 1.10E-08 |
| 39 | 26224 | 26778 | - | 184 | 21271 | 4.59 | putative tail fiber assembly protein | *Erwinia* phage pEp_SNUABM_01  28.1 | QEQ94897.1 | 2.40E-114 |
| 40 | 26788 | 27993 | - | 401 | 42586.3 | 5.11 | conserved tail fiber protein | *Cronobacter* phage vB_CsaM_GAP31  68.9 | AFC21407.1 | 4.10E-113 |
| 41 | 28005 | 28637 | - | 210 | 23338.2 | 4.93 | hypothetical protein | *Salmonella* phage SSE121  95.7 | AFU63682.1 | 8.00E-261 |
| 42 | 28648 | 30141 | - | 497 | 54083.9 | 4.41 | hypothetical protein GAP31_224 | *Cronobacter* phage vB_CsaM_GAP31  93.8 | AFC21405.1 | 0.00E+00 |
| 43 | 30250 | 32340 | - | 696 | 75027.5 | 5.39 | putative fusion protein | *Cronobacter* phage vB_CsaM_GAP31  93.7 | AFC21404.1 | 0.00E+00 |
| 44 | 32398 | 35187 | - | 929 | 105956.4 | 4.43 | conserved hypothetical protein | *Salmonella* phage PVPSE1  96.4 | ADP02445.1 | 3.10E-111 |
| 45 | 35187 | 35861 | - | 224 | 23543.4 | 5.13 | hypothetical protein GAP31_221 | *Cronobacter* phage vB_CsaM_GAP31  89.7 | AFC21402.1 | 7.70E-117 |
| 46 | 35872 | 36489 | - | 205 | 23813.5 | 4.36 | hypothetical protein | *Escherichia* phage 4MG  98 | AGZ17524.1 | 6.50E-128 |
| 47 | 36499 | 37200 | - | 233 | 25136.7 | 4.96 | putative baseplate assembly protein | *Escherichia* phage 4MG  94 | AGZ17525.1 | 3.10E-168 |
| 48 | 37200 | 38189 | - | 329 | 36370.7 | 9.37 | hypothetical protein | *Escherichia* phage 4MG  97.6 | AGZ17526.1 | 3.80E-60 |
| 49 | 38193 | 38543 | - | 116 | 13477.9 | 3.97 | hypothetical protein | *Escherichia* phage 4MG  98.3 | AGZ17527.1 | 4.70E-152 |
| 50 | 38543 | 39415 | - | 290 | 32005.5 | 5.96 | hypothetical protein | *Escherichia* phage 4MG  95.2 | AGZ17528.1 | 0.00E+00 |
| 51 | 39497 | 41887 | - | 796 | 87529.5 | 10.19 | conserved hypothetical membrane protein | *Salmonella* phage PVPSE1  93.3 | ADP02452.1 | 2.40E-34 |
| 52 | 41941 | 42183 | - | 80 | 9481.5 | 7.34 | conserved hypothetical protein | *Salmonella* phage PVPSE1  90 | ADP02453.1 | 8.00E-82 |
| 53 | 42219 | 42692 | - | 157 | 17275.9 | 4.39 | hypothetical protein GAP31_213 | *Cronobacter* phage vB_CsaM_GAP31  98.1 | AFC21394.1 | 7.30E-83 |
| 54 | 42767 | 43240 | - | 157 | 16890.1 | 4.39 | hypothetical protein | *Salmonella* phage SSE121  96.2 | AFU63669.1 | 1.10E-243 |
| 55 | 43244 | 44656 | - | 470 | 50142.1 | 4.81 | tail sheath protein | *Escherichia* phage 4MG  96.4 | AGZ17533.1 | 1.90E-87 |
| 56 | 44784 | 45314 | - | 176 | 19741 | 4.12 | hypothetical protein GAP31_210 | *Cronobacter* phage vB_CsaM_GAP31  90.3 | AFC21391.1 | 2.50E-74 |
| 57 | 45314 | 45748 | - | 144 | 16185.2 | 5.23 | hypothetical protein GAP31_209 | *Cronobacter* phage vB_CsaM_GAP31  92.4 | AFC21390.1 | 3.60E-82 |
| 58 | 45748 | 46224 | - | 158 | 17625.8 | 9.93 | hypothetical protein GAP31_208 | *Cronobacter* phage vB_CsaM_GAP31  93 | AFC21389.1 | 4.20E-92 |
| 59 | 46290 | 46808 | - | 172 | 19410.1 | 4.98 | hypothetical protein | *Salmonella* phage SSE121  93 | AFU63664.1 | 1.20E-57 |
| 60 | 46942 | 47346 | + | 134 | 14696.9 | 8.64 | putative membrane protein | *Cronobacter* phage vB_CsaM_GAP31  80.6 | AFC21387.1 | 1.10E-15 |
| 61 | 47368 | 47754 | - | 128 | 12187.6 | 6.73 | hypothetical protein pEpSNUABM01_092 | *Erwinia* phage pEp_SNUABM_01  44.5 | QEQ94918.1 | 5.60E-102 |
| 62 | 47768 | 48412 | - | 214 | 24463 | 4.09 | hypothetical protein | *Salmonella* phage SSE121  82.2 | AFU63661.1 | 2.60E-299 |
| 63 | 48449 | 51070 | - | 873 | 88855.4 | 4.02 | tail fiber protein | *Salmonella* phage SSE121  84.8 | AFU63660.1 | 3.30E-92 |
| 64 | 51080 | 51613 | - | 177 | 19419 | 6.55 | hypothetical protein | *Salmonella* phage SSE121  97.2 | AFU63659.1 | 7.30E-189 |
| 65 | 51746 | 52759 | - | 337 | 38497.9 | 5.1 | hypothetical protein | *Salmonella* phage SSE121  97.3 | AFU63658.1 | 4.10E-69 |
| 66 | 52781 | 53200 | - | 139 | 14607.5 | 4.14 | hypothetical protein | *Salmonella* phage SSE121  97.8 | AFU63657.1 | 4.40E-170 |
| 67 | 53219 | 54223 | - | 334 | 36823.9 | 4.3 | hypothetical protein | *Salmonella* phage SSE121  98.2 | AFU63656.1 | 8.70E-108 |
| 68 | 54220 | 54849 | - | 209 | 22998.8 | 4.29 | hypothetical protein | *Salmonella* phage SSE121  98.1 | AFU63655.1 | 2.70E-280 |
| 69 | 54916 | 56433 | - | 505 | 55120.9 | 5.07 | hypothetical protein | *Salmonella* phage SSE121  99 | AFU63654.1 | 8.60E-292 |
| 70 | 56482 | 57942 | - | 486 | 55535.3 | 5.21 | terminase large subunit | *Salmonella* phage PVPSE1  99.2 | ADP02471.1 | 5.30E-44 |
| 71 | 58082 | 58372 | - | 96 | 10421.1 | 10.02 | hypothetical protein | *Salmonella* phage SSE121  100 | AFU63652.1 | 2.40E-47 |
| 72 | 58369 | 58770 | - | 133 | 15155 | 5.24 | hypothetical protein | *Salmonella* phage SSE121  96.2 | AFU63651.1 | 4.80E-202 |
| 73 | 58767 | 60209 | - | 480 | 55094.5 | 5.01 | hyphothetical protein | *Escherichia* phage 4MG  72.6 | AGZ17551.1 | 6.90E-20 |
| 74 | 60669 | 61301 | - | 210 | 22994.8 | 8.93 | hypothetical protein GAP32_002 | *Cronobacter* phage vB_CsaM_GAP32  35.1 | AFC21449.1 | 3.10E-23 |
| 75 | 61360 | 61626 | - | 88 | 9800.5 | 11.49 | / | / | / | 9.30E-07 |
| 76 | 61613 | 61789 | - | 58 | 6195.9 | 4.81 | hypothetical protein CPT_Mydo_216 | *Proteus* phage Mydo  91.4 | AZF87772.1 | 4.50E-33 |
| 77 | 62278 | 62649 | + | 123 | 13975.9 | 6.34 | / | / | / | 9.70E-51 |
| 78 | 63037 | 63216 | - | 59 | 6982.3 | 10.23 | hypothetical protein PBI_RHYNO_33 | *Mycobacterium* phage RhynO  46.3 | AHJ88691.1 | 1.70E-106 |
| 79 | 63321 | 63722 | - | 133 | 15512.8 | 8.56 | HNH endonuclease | *Salmonella* phage Meda  56.8 | AXY86342.1 | 5.90E-93 |
| 80 | 64162 | 64530 | - | 122 | 14049.8 | 5.37 | hypothetical protein | *Salmonella* phage SSE121  88.4 | AFU63644.1 | 4.40E-23 |
| 81 | 64550 | 64723 | + | 57 | 6518.6 | 5.73 | / | / | / | 1.00E-51 |
| 82 | 65702 | 66349 | - | 215 | 23752.8 | 5.22 | hypothetical protein | *Salmonella* phage SSE121  97.7 | AFU63883.1 | 1.20E-21 |
| 83 | 66427 | 67038 | - | 203 | 21875.5 | 4.81 | hypothetical protein | *Salmonella* phage SSE121  98 | AFU63882.1 | 2.10E-22 |
| 84 | 67214 | 67402 | - | 62 | 6376.3 | 3.86 | hypothetical protein | *Salmonella* phage SSE121  95.2 | AFU63880.1 | 4.60E-41 |
| 85 | 67608 | 67937 | - | 109 | 11979.7 | 11.39 | hypothetical protein SP41_143 | *Salmonella* phage 41  100 | AKJ73432.1 | 3.30E-32 |
| 86 | 68008 | 68175 | - | 55 | 6494.4 | 8.95 | hypothetical protein | *Salmonella* phage SSE121  89.1 | AFU63878.1 | 5.20E-92 |
| 87 | 68364 | 68603 | - | 79 | 8629.9 | 9.86 | hypothetical protein | *Salmonella* phage SSE121  100 | AFU63877.1 | 2.30E-32 |
| 88 | 69023 | 69268 | - | 81 | 9255.1 | 4.44 | conserved hypothetical protein | *Salmonella* phage PVPSE1  100 | ADP02487.1 | 8.1e-310 |
| 89 | 69348 | 69554 | - | 68 | 7825.6 | 4.66 | conserved hypothetical protein | *Salmonella* phage PVPSE1  98.5 | ADP02488.1 | 3.00E-85 |
| 90 | 69538 | 70029 | - | 163 | 19140.6 | 10.12 | hypothetical protein | *Salmonella* phage SSE121  99.4 | AFU63873.1 | 4.80E-135 |
| 91 | 70022 | 70213 | - | 63 | 6909.9 | 4.66 | hypothetical protein | *Salmonella* phage PVPSE1  100 | ADP02490.1 | 5.30E-53 |
| 92 | 70349 | 72034 | - | 561 | 63784.4 | 4.79 | nicotinamide phosphoribosyl transferase | *Salmonella* phage SSE121  92.6 | AFU63871.1 | 5.20E-48 |
| 93 | 72088 | 72621 | - | 177 | 20200.4 | 4.46 | hyphothetical protein | *Escherichia* phage 4MG  81.2 | AGZ17566.1 | 1.40E-39 |
| 94 | 72621 | 73421 | - | 266 | 29142.2 | 6.52 | putative phosphoribosyl pyrophosphate synthetase | *Escherichia* phage 4MG  88 | AGZ17567.1 | 4.30E-46 |
| 95 | 73423 | 73740 | - | 105 | 12269.9 | 9.73 | hypothetical protein GAP31_179 | *Cronobacter* phage vB_CsaM_GAP31  96.2 | AFC21360.1 | 6.20E-13 |
| 96 | 74014 | 74337 | + | 107 | 12260.2 | 8.06 | hypothetical protein | *Salmonella* phage SSE121  90.7 | AFU63868.1 | 1.20E-111 |
| 97 | 74500 | 74733 | + | 77 | 9002.4 | 8.7 | hypothetical protein | *Salmonella* phage SSE121  100 | AFU63867.1 | 1.30E-90 |
| 98 | 74730 | 75017 | + | 95 | 11225 | 9.51 | hypothetical protein | *Salmonella* phage SSE121  93.7 | AFU63866.1 | 2.80E-58 |
| 99 | 75014 | 75298 | + | 94 | 10969.1 | 5.53 | F-box domain protein | *Acinetobacter* phage vB_AbaM_ME3  51.4 | AND75426.1 | 8.60E-22 |
| 100 | 75276 | 75869 | + | 197 | 22477.4 | 7.55 | putative phosphoesterase | *Cronobacter* phage vB_CsaM_GAP31  92.9 | AFC21355.1 | 1.10E-165 |
| 101 | 75866 | 76726 | + | 286 | 31778.5 | 6.15 | hypothetical protein pangalan_129 | *Escherichia* phage pangalan  57.5 | QHR73363.1 | 6.30E-60 |
| 102 | 76716 | 77084 | + | 122 | 14106 | 6.24 | hypothetical protein CPT_Mydo_171 | *Proteus* phage Mydo  89 | AZF87746.1 | 1.30E-72 |
| 103 | 77068 | 77265 | + | 65 | 7880 | 8.97 | hypothetical protein Ro1_00141 | *Raoultella* phage Ro1  81.5 | AUE23367.1 | 6.40E-137 |
| 104 | 77234 | 78178 | + | 314 | 35847.6 | 5.26 | hypothetical protein | *Salmonella* phage SSE121  88.1 | AFU63862.1 | 4.60E-212 |
| 105 | 78175 | 78519 | + | 114 | 13530.2 | 4.92 | hypothetical protein GAP31_170 | *Cronobacter* phage vB_CsaM_GAP31  93 | AFC21351.1 | 6.90E-75 |
| 106 | 78529 | 78936 | + | 135 | 15616.4 | 6.8 | hypothetical protein | *Salmonella* phage SSE121  100 | AFU63860.1 | 1.60E-64 |
| 107 | 78946 | 79719 | + | 257 | 29322 | 5.38 | NAD-dependent protein deacetylase of SIR2 family | *Salmonella* phage SSE121  89.5 | AFU63859.1 | 4.90E-128 |
| 108 | 79721 | 80035 | + | 104 | 12390.3 | 9.5 | / | / | / | 4.10E-38 |
| 109 | 80035 | 81330 | + | 431 | 49294.2 | 5.04 | DNA ligase | *Salmonella* phage SSE121  83.8 | AFU63858.1 | 4.10E-50 |
| 110 | 81330 | 81776 | + | 148 | 16624.7 | 4.7 | hypothetical protein | *Salmonella* phage SSE121  90.5 | AFU63857.1 | 1.50E-29 |
| 111 | 81776 | 82147 | + | 123 | 13988.3 | 9.04 | putative membrane protein | *Cronobacter* phage vB_CsaM_GAP31  92.7 | AFC21346.1 | 5.70E-137 |
| 112 | 82157 | 82849 | + | 230 | 25763.3 | 5 | conserved hypothetical protein | *Salmonella* phage PVPSE1  99.6 | ADP02508.1 | 3.60E-28 |
| 113 | 82872 | 83144 | + | 90 | 10737.7 | 10.44 | hypothetical protein | *Salmonella* phage PVPSE1  97.5 | ADP02509.1 | 1.90E-32 |
| 114 | 83213 | 83527 | + | 104 | 11934.5 | 8.94 | hypothetical protein | *Salmonella* phage SSE121  100 | AFU63853.1 | 3.90E-101 |
| 115 | 83527 | 83730 | + | 67 | 7470 | 10.15 | hypothetical protein | *Salmonella* phage SSE121  95.5 | AFU63852.1 | 2.00E-82 |
| 116 | 83871 | 84761 | + | 296 | 32619.2 | 8.79 | hypothetical protein | *Salmonella* phage SSE121  99.7 | AFU63850.1 | 7.70E-224 |
| 117 | 84825 | 85019 | + | 64 | 6964.9 | 8.22 | hypothetical membrane protein | *Salmonella* phage PVPSE1  100 | ADP02514.1 | 3.40E-52 |
| 118 | 85029 | 85232 | + | 67 | 7760.2 | 10.44 | hypothetical membrane protein | *Salmonella* phage PVPSE1  100 | ADP02515.1 | 9.30E-95 |
| 119 | 85216 | 85740 | + | 174 | 20227.2 | 8.24 | conserved hypothetical protein | *Salmonella* phage PVPSE1  98.3 | ADP02516.1 | 3.60E-99 |
| 120 | 85737 | 86174 | + | 145 | 16787.3 | 9.24 | hypothetical protein | *Salmonella* phage SSE121  98.6 | AFU63846.1 | 1.80E-192 |
| 121 | 86183 | 87304 | + | 373 | 42806.6 | 7.22 | hypothetical protein | *Salmonella* phage SSE121  98.9 | AFU63845.1 | 4.90E-27 |
| 122 | 87301 | 87618 | + | 105 | 11633.2 | 3.72 | hypothetical protein | *Salmonella* phage SSE121  99 | AFU63844.1 | 1.30E-116 |
| 123 | 87680 | 88213 | + | 177 | 19819.7 | 9.18 | hypothetical protein | *Salmonella* phage SSE121  98.9 | AFU63843.1 | 4.90E-116 |
| 124 | 88258 | 88788 | + | 176 | 19991.7 | 5.31 | EndoVII packaging and recombination endonuclease | *Salmonella* phage PVPSE1  99.4 | ADP02521.1 | 1.40E-32 |
| 125 | 88945 | 89919 | + | 324 | 37512.6 | 6.38 | hypothetical protein | *Salmonella* phage SSE121  99.4 | AFU63840.1 | 5.00E-35 |
| 126 | 89951 | 90136 | + | 61 | 7150 | 4.03 | hypothetical protein | *Salmonella* phage SSE121  98.4 | AFU63839.1 | 4.70E-169 |
| 127 | 90136 | 90741 | + | 201 | 22907.5 | 4.88 | hypothetical protein | *Salmonella* phage SSE121  98.5 | AFU63838.1 | 8.40E-87 |
| 128 | 90734 | 91336 | + | 200 | 23073 | 4.8 | hypothetical protein | *Salmonella* phage SSE121  98.5 | AFU63837.1 | 7.00E-39 |
| 129 | 91336 | 91530 | + | 64 | 7468.6 | 8.21 | hypothetical protein | *Salmonella* phage SSE121  100 | AFU63836.1 | 0.00E+00 |
| 130 | 91527 | 91850 | + | 107 | 12015.8 | 4.32 | hypothetical protein | *Escherichia* phage 4MG  71.4 | AGZ17603.1 | 1.70E-204 |
| 131 | 91850 | 92815 | + | 321 | 36145.4 | 5.01 | putative thymidylate synthase | *Escherichia* phage 4MG  89.7 | AGZ17604.1 | 8.20E-47 |
| 132 | 92898 | 93374 | + | 158 | 18024.2 | 5.02 | hypothetical protein | *Salmonella* phage SSE121  99.4 | AFU63832.1 | 5.60E-40 |
| 133 | 93374 | 93604 | + | 76 | 9084.7 | 4.19 | hypothetical protein | *Salmonella* phage SSE121  100 | AFU63831.1 | 9.50E-66 |
| 134 | 93614 | 95929 | + | 771 | 87186.7 | 6.3 | ribonucleotide reductase of class Ia (aerobic) alpha subunit | *Salmonella* phage SSE121  98.2 | AFU63830.1 | 7.00E-130 |
| 135 | 95968 | 97056 | + | 362 | 41802.3 | 4.26 | ribonucleoside diphosphate reductase beta chain | *Cronobacter* phage vB_CsaM_GAP31  96.7 | AFC21322.1 | 1.90E-144 |
| 136 | 97066 | 97419 | + | 117 | 13122.9 | 4.84 | hypothetical protein | *Salmonella* phage SSE121  71.3 | AFU63828.1 | 1.30E-119 |
| 137 | 97416 | 97682 | + | 88 | 10060.4 | 5.85 | glutaredoxin 1 | *Cronobacter* phage vB_CsaM_GAP31  85.2 | AFC21320.1 | 7.80E-42 |
| 138 | 97904 | 98329 | + | 141 | 15791 | 4.26 | hypothetical protein | *Salmonella* phage SSE121  97.2 | AFU63823.1 | 2.10E-79 |
| 139 | 98348 | 99058 | + | 236 | 25326.5 | 9.22 | hypothetical protein | *Salmonella* phage SSE121  97.5 | AFU63822.1 | 1.90E-172 |
| 140 | 99070 | 99858 | + | 262 | 29323.1 | 8.64 | hypothetical protein | *Salmonella* phage SSE121  99.6 | AFU63821.1 | 2.70E-131 |
| 141 | 99869 | 100501 | + | 210 | 23881.8 | 8.83 | hypothetical protein | *Salmonella* phage SSE121  100 | AFU63820.1 | 3.80E-18 |
| 142 | 100530 | 100796 | + | 88 | 10150.5 | 8.42 | hypothetical protein | *Salmonella* phage SSE121  94.3 | AFU63819.1 | 8.00E-50 |
| 143 | 100846 | 101295 | + | 149 | 17620.9 | 5.31 | hypothetical protein | *Salmonella* phage SSE121  95.3 | AFU63818.1 | 9.40E-35 |
| 144 | 101297 | 102226 | + | 309 | 34341.6 | 4.44 | ClpP ATP-dependent protease subunit | *Salmonella* phage PVPSE1  99.7 | ADP02547.1 | 3.10E-71 |
| 145 | 102226 | 102906 | + | 226 | 25731.8 | 5.49 | hypothetical protein | *Salmonella* phage SSE121  99.6 | AFU63816.1 | 2.60E-52 |
| 146 | 103068 | 103556 | + | 162 | 18637.1 | 10.35 | putative HNH endonuclease | *Pectobacterium* phage vB_PatM_CB7  40 | ARB11583.1 | 4.40E-37 |
| 147 | 103605 | 103880 | + | 91 | 11075.7 | 8.64 | hypothetical protein | *Salmonella* phage SSE121  100 | AFU63814.1 | 4.10E-242 |
| 148 | 103877 | 104086 | + | 69 | 8021.1 | 5.68 | hypothetical protein | *Salmonella* phage SSE121  100 | AFU63813.1 | 6.40E-36 |
| 149 | 104083 | 104475 | + | 130 | 15236.9 | 4.18 | conserved hypothetical protein | *Salmonella* phage PVPSE1  98.4 | ADP02552.1 | 3.90E-31 |
| 150 | 104502 | 104819 | + | 105 | 11360.8 | 4.5 | hypothetical protein | *Salmonella* phage SSE121  97.1 | AFU63811.1 | 2.60E-83 |
| 151 | 104816 | 105082 | + | 88 | 10442.9 | 8.2 | hypothetical protein GAP31_121 | *Cronobacter* phage vB_CsaM_GAP31  81.6 | AFC21303.1 | 3.80E-24 |
| 152 | 105092 | 106345 | + | 417 | 46484.5 | 6.46 | tRNA nucleotidyltransferase | *Salmonella* phage SSE121  98.3 | AFU63809.1 | 4.70E-52 |
| 153 | 106356 | 106562 | + | 68 | 8045.2 | 9.22 | hypothetical protein | *Salmonella* phage SSE121  100 | AFU63808.1 | 3.30E-67 |
| 154 | 106573 | 106791 | + | 72 | 8022.8 | 4.74 | hypothetical protein | *Salmonella* phage SSE121  98.6 | AFU63807.1 | 1.90E-192 |
| 155 | 106788 | 107279 | + | 163 | 18562 | 5.18 | hypothetical protein KB57_157 | *Klebsiella* phage vB_KpnM_KB57  89 | ALM02544.1 | 4.00E-33 |
| 156 | 107281 | 107463 | + | 60 | 6814.8 | 4.98 | hypothetical protein | *Salmonella* phage SSE121  100 | AFU63805.1 | 1.40E-28 |
| 157 | 107463 | 107795 | + | 110 | 12435 | 9.14 | hypothetical protein | *Salmonella* phage SSE121  98.2 | AFU63804.1 | 4.30E-33 |
| 158 | 107805 | 108167 | + | 120 | 14090 | 6.78 | hypothetical protein | *Salmonella* phage SSE121  98.3 | AFU63803.1 | 2.70E-59 |
| 159 | 108176 | 109237 | + | 353 | 41167.7 | 6.08 | hypothetical protein | *Salmonella* phage SSE121  99.7 | AFU63802.1 | 5.50E-08 |
| 160 | 109230 | 109442 | + | 70 | 7942.3 | 8.47 | hypothetical membrane protein | *Salmonella* phage PVPSE1  100 | ADP02563.1 | 4.00E-76 |
| 161 | 109429 | 109605 | + | 58 | 7214.1 | 4.2 | hypothetical protein | *Salmonella* phage PVPSE1  96.6 | ADP02564.1 | 3.40E-58 |
| 162 | 109602 | 109829 | + | 75 | 8028.3 | 7.15 | hypothetical protein | *Salmonella* phage PVPSE1  80 | ADP02565.1 | 1.00E-74 |
| 163 | 109837 | 110340 | + | 167 | 18143.8 | 8.68 | hypothetical protein CR3_255 | *Cronobacter* phage CR3  72.8 | AFH21420.1 | 2.10E-54 |
| 164 | 110337 | 110489 | + | 50 | 5304.1 | 9.87 | hypothetical protein CR3_255 | *Cronobacter* phage CR3  64.6 | AFH21420.1 | 9.80E-32 |
| 165 | 110568 | 111056 | + | 162 | 19183.1 | 7.59 | hypothetical protein | *Salmonella* phage SSE121  87 | AFU63798.1 | 1.00E-38 |
| 166 | 111126 | 111461 | + | 111 | 13051.9 | 9.42 | hypothetical protein | *Salmonella* phage PVPSE1  100 | ADP02567.1 | 1.10E-33 |
| 167 | 111446 | 111940 | + | 164 | 18573.5 | 7.66 | hypothetical protein | *Salmonella* phage PVPSE1  86.6 | ADP02568.1 | 2.60E-39 |
| 168 | 111966 | 112271 | + | 101 | 11775.5 | 5.17 | hypothetical protein | *Salmonella* phage PVPSE1  95 | ADP02569.1 | 4.80E-70 |
| 169 | 112395 | 112604 | + | 69 | 7904 | 5.12 | hypothetical protein | *Salmonella* phage PVPSE1  100 | ADP02570.1 | 6.50E-20 |
| 170 | 112753 | 113010 | + | 85 | 10080.5 | 10.62 | conserved hypothetical protein | *Salmonella* phage PVPSE1  86 | ADP02572.1 | 5.50E-11 |
| 171 | 113007 | 113225 | + | 72 | 8411.1 | 8.49 | hypothetical membrane protein | *Salmonella* phage PVPSE1  98.6 | ADP02573.1 | 2.20E-28 |
| 172 | 113225 | 113479 | + | 84 | 10352.5 | 6.97 | hypothetical protein | *Salmonella* phage PVPSE1  96.4 | ADP02574.1 | 2.40E-57 |
| 173 | 113519 | 113677 | + | 52 | 5611.9 | 8.8 | / | / | / | 2.60E-41 |
| 174 | 113898 | 114320 | + | 140 | 15849.2 | 6.78 | hypothetical protein BIS47_172 | *Klebsiella* phage vB_KpnM_BIS47  87.9 | ARB12676.1 | 6.20E-22 |
| 175 | 114805 | 114963 | + | 52 | 6145.1 | 4.46 | hypothetical protein | *Salmonella* phage SSE121  86.5 | AFU63784.1 | 6.10E-60 |
| 176 | 115152 | 115358 | - | 68 | 8243.9 | 10.08 | hypothetical protein KB57_185 | *Klebsiella* phage vB_KpnM_KB57  63.2 | ALM02572.1 | 1.90E-56 |
| 177 | 115427 | 115582 | - | 51 | 5646.8 | 11.7 | / | / | / | 3.90E-26 |
| 178 | 116329 | 116592 | - | 87 | 9770.5 | 8.48 | hypothetical protein | *Salmonella* phage PVPSE1  96.9 | ADP02576.1 | 2.70E-48 |
| 179 | 116585 | 116950 | - | 121 | 13713.4 | 4.54 | hyphothetical protein | *Escherichia* phage 4MG  90.1 | AGZ17677.1 | 1.80E-30 |
| 180 | 117001 | 117309 | - | 102 | 11929.2 | 5.16 | hypothetical protein CR8_160 | *Cronobacter* phage CR8  76.5 | AIA64690.1 | 1.40E-51 |
| 181 | 117392 | 117631 | - | 79 | 9029.5 | 10.46 | hypothetical protein CPT_Mydo_075 | *Proteus* phage Mydo  70.3 | AZF87650.1 | 3.70E-61 |
| 182 | 117820 | 118122 | - | 100 | 11279.8 | 5.77 | / | / | / | 2.60E-85 |
| 183 | 118210 | 118542 | - | 110 | 12631.9 | 4.01 | hypothetical protein GAP31_063 | *Cronobacter* phage vB_CsaM_GAP31  98.2 | AFC21244.1 | 3.80E-137 |
| 184 | 118597 | 118938 | - | 113 | 12742.3 | 4.67 | hypothetical protein | *Salmonella* phage SSE121  95.6 | AFU63778.1 | 6.40E-28 |
| 185 | 119350 | 119523 | - | 57 | 6592.6 | 6.48 | hypothetical protein | *Salmonella* phage SSE121  96.5 | AFU63775.1 | 6.30E-11 |
| 186 | 119749 | 120033 | - | 94 | 11064.7 | 10.6 | hypothetical protein | *Salmonella* phage SSE121  100 | AFU63774.1 | 1.90E-97 |
| 187 | 120378 | 120581 | - | 67 | 8196.2 | 10.53 | hypothetical protein | *Salmonella* phage SSE121  97 | AFU63773.1 | 1.90E-158 |
| 188 | 120598 | 120942 | - | 114 | 13750.8 | 9.94 | hypothetical protein | *Salmonella* phage SSE121  84.1 | AFU63772.1 | 2.30E-17 |
| 189 | 120939 | 121319 | - | 126 | 14543.5 | 6.79 | hypothetical protein CPT_Mydo_060 | *Proteus* phage Mydo  88 | AZF87635.1 | 7.00E-74 |
| 190 | 121387 | 121851 | - | 154 | 17799.2 | 10.17 | hypothetical protein CPT_Mydo_059 | *Proteus* phage Mydo  96.8 | AZF87634.1 | 1.10E-71 |
| 191 | 121926 | 122711 | - | 261 | 29202.3 | 9.78 | hypothetical protein BIS47_201 | *Klebsiella* phage vB_KpnM_BIS47  87.7 | ARB12705.1 | 6.80E-51 |
| 192 | 122775 | 123122 | - | 115 | 12781.7 | 10.85 | hypothetical protein | *Escherichia* phage 4MG  56.5 | AGZ17700.1 | 1.90E-110 |
| 193 | 123394 | 123576 | + | 60 | 6734.6 | 6.23 | hypothetical protein | *Salmonella* phage PVPSE1  55.4 | ADP02597.1 | 3.80E-31 |
| 194 | 124363 | 124854 | - | 163 | 19467.9 | 9.02 | hypothetical protein | *Salmonella* phage SSE121  98.2 | AFU63764.1 | 6.80E-68 |
| 195 | 124978 | 125823 | - | 281 | 32222.9 | 4.42 | hypothetical protein | *Salmonella* phage SSE121  100 | AFU63763.1 | 1.60E-32 |
| 196 | 125949 | 126110 | - | 53 | 6681.8 | 9.61 | hypothetical protein | *Salmonella* phage SSE121  84.8 | AFU63762.1 | 1.50E-65 |
| 197 | 126110 | 126520 | - | 136 | 15558.5 | 7.59 | hypothetical protein SP40_124 | *Salmonella* phage 40  97.8 | AKJ73565.1 | 1.00E-255 |
| 198 | 126480 | 126869 | - | 129 | 15057.2 | 4.69 | putative carbohydrate binding domain protein | *Salmonella* phage 40  98.4 | AKJ73564.1 | 5.40E-24 |
| 199 | 126866 | 127201 | - | 111 | 12628.3 | 4.37 | hypothetical protein | *Salmonella* phage SSE121  92.8 | AFU63759.1 | 1.60E-144 |
| 200 | 127198 | 127845 | - | 215 | 24359.8 | 6.81 | hypothetical protein | *Salmonella* phage SSE121  94 | AFU63758.1 | 4.60E-74 |
| 201 | 127857 | 128069 | - | 70 | 7888.1 | 8.03 | hypothetical protein | *Salmonella* phage SSE121  91.4 | AFU63757.1 | 3.50E-67 |
| 202 | 128066 | 128440 | - | 124 | 13747.4 | 4.84 | hypothetical protein | *Salmonella* phage SSE121  96.8 | AFU63756.1 | 5.70E-27 |
| 203 | 128713 | 129009 | - | 98 | 11156.8 | 9.45 | hypothetical protein GAP31_035 | *Cronobacter* phage vB_CsaM_GAP31  72.4 | AFC21214.1 | 5.70E-32 |
| 204 | 129006 | 129395 | - | 129 | 14852 | 4.79 | hypothetical protein | *Salmonella* phage SSE121  93.7 | AFU63754.1 | 5.30E-79 |
| 205 | 129379 | 130866 | - | 495 | 57030.7 | 8.13 | hypothetical protein | *Salmonella* phage SSE121  88.1 | AFU63753.1 | 6.70E-71 |
| 206 | 130875 | 131132 | - | 85 | 9628.2 | 7.73 | hypothetical protein GAP31_032 | *Cronobacter* phage vB_CsaM_GAP31  56.8 | AFC21211.1 | 7.40E-29 |
| 207 | 131132 | 132007 | - | 291 | 33183.6 | 7.45 | hypothetical protein | *Salmonella* phage SSE121  97.6 | AFU63751.1 | 3.40E-38 |
| 208 | 132135 | 133154 | + | 339 | 40235.8 | 6.91 | hypothetical protein | *Salmonella* phage SSE121  43.1 | AFU63750.1 | 1.30E-232 |
| 209 | 133118 | 133624 | + | 168 | 19041.9 | 6.97 | hypothetical protein | *Salmonella* phage SSE121  80.7 | AFU63749.1 | 1.60E-28 |
| 210 | 133621 | 133836 | + | 71 | 8479.7 | 9.14 | Phi92_gp013 | *Enterobacteria* phage phi92  75 | CBY99441.1 | 5.50E-49 |
| 211 | 134054 | 134263 | + | 69 | 8036 | 10.1 | hypothetical protein GAP31_028 | *Cronobacter* phage vB_CsaM_GAP31  91.3 | AFC21206.1 | 4.40E-37 |
| 212 | 134265 | 134774 | + | 169 | 19910.7 | 9.71 | hypothetical protein | *Salmonella* phage SSE121  81.7 | AFU63747.1 | 4.70E-27 |
| 213 | 134767 | 135147 | + | 126 | 15171 | 4.76 | hypothetical protein | *Salmonella* phage SSE121  96.8 | AFU63746.1 | 6.50E-29 |
| 214 | 135177 | 135377 | + | 66 | 7745.7 | 5.08 | hypothetical protein | *Salmonella* phage PVPSE1  100 | ADP02620.1 | 1.00E-87 |
| 215 | 135367 | 136026 | + | 219 | 25658 | 8.52 | hypothetical protein CBB_347 | *Pectobacterium* phage CBB  37.3 | AMM43910.1 | 2.00E-29 |
| 216 | 136013 | 137221 | + | 402 | 45219.9 | 4.85 | hypothetical protein | *Salmonella* phage SSE121  98.3 | AFU63745.1 | 3.20E-23 |
| 217 | 137287 | 137481 | + | 64 | 7587.3 | 4.35 | hypothetical protein GAP31_021 | *Cronobacter* phage vB_CsaM_GAP31  88.9 | AFC21199.1 | 8.00E-31 |
| 218 | 137474 | 137764 | + | 96 | 11062.4 | 6.78 | hypothetical protein | *Salmonella* phage SSE121  94.8 | AFU63743.1 | 1.80E-36 |
| 219 | 137758 | 137961 | + | 67 | 7757.6 | 4.15 | hypothetical protein | *Salmonella* phage SSE121  95.5 | AFU63742.1 | 2.20E-55 |
| 220 | 137961 | 138137 | + | 58 | 6972.1 | 9.29 | hypothetical protein | *Salmonella* phage SSE121  100 | AFU63741.1 | 2.70E-199 |
| 221 | 138134 | 138310 | + | 58 | 6900.6 | 4.02 | hypothetical protein | *Salmonella* phage SSE121  94.8 | AFU63740.1 | 9.60E-20 |
| 222 | 138307 | 138807 | + | 166 | 18298 | 7.24 | hypothetical protein SP40_91 | *Salmonella* phage 40  92.7 | AKJ73532.1 | 1.30E-22 |
| 223 | 138800 | 139003 | + | 67 | 7412.5 | 7.7 | hypothetical protein | *Escherichia* phage 4MG  90.8 | AGZ17736.1 | 1.90E-38 |
| 224 | 139003 | 139185 | + | 60 | 7126.5 | 9.74 | hypothetical protein GAP31_012 | *Cronobacter* phage vB_CsaM_GAP31  86.7 | AFC21190.1 | 6.90E-60 |
| 225 | 139182 | 139385 | + | 67 | 7606.5 | 9.09 | hypothetical protein | *Salmonella* phage SSE121  97 | AFU63735.1 | 7.00E-80 |
| 226 | 139378 | 139596 | + | 72 | 8398.5 | 8.22 | hypothetical protein | *Salmonella* phage SSE121  100 | AFU63734.1 | 1.60E-28 |
| 227 | 139589 | 139954 | + | 121 | 13279.1 | 5.69 | hypothetical protein GAP31_008 | *Cronobacter* phage vB_CsaM_GAP31  86.8 | AFC21186.1 | 5.50E-49 |
| 228 | 139947 | 140984 | + | 345 | 38897.2 | 4.97 | hypothetical protein | *Salmonella* phage SSE121  98.8 | AFU63731.1 | 4.40E-37 |
| 229 | 140984 | 141163 | + | 59 | 6707.6 | 5.01 | hypothetical protein | *Salmonella* phage SSE121  86.7 | AFU63730.1 | 4.70E-27 |
| 230 | 141138 | 141335 | + | 65 | 7257 | 4.05 | putative integrase | *Salmonella* phage 40  76.7 | AKJ73522.1 | 6.50E-29 |
| 231 | 141361 | 141726 | + | 121 | 12623.9 | 4.6 | hypothetical protein | *Salmonella* phage SSE121  99.2 | AFU63729.1 | 1.00E-87 |
| 232 | 141727 | 142371 | + | 214 | 25004.6 | 9.19 | hypothetical protein BIS47_240 | *Klebsiella* phage vB_KpnM_BIS47  54.3 | ARB12744.1 | 2.70E-199 |
| 233 | 142455 | 142892 | + | 145 | 16999.3 | 9.82 | hypothetical protein SP40_78 | *Salmonella* phage 40  97.9 | AKJ73519.1 | 9.60E-20 |
